# A Versatile Nanoluciferase Reporter Reveals Structural Properties Associated with a Highly Efficient, N-Terminal Legionella pneumophila Type IV Secretion Translocation Signal

**DOI:** 10.1128/spectrum.02338-22

**Published:** 2023-02-23

**Authors:** Yoon-Suk Kang, James E. Kirby

**Affiliations:** a Department of Pathology, Beth Israel Deaconess Medical Center, Boston, Massachusetts, USA; b Harvard Medical School, Boston, Massachusetts, USA; Emory University School of Medicine

**Keywords:** type IV secretion system, gene reporters, nanoluciferase, protein translocation, split GFP, translocation signal

## Abstract

Many Gram-negative pathogens rely on type IV secretion systems (T4SS) for infection. One limitation has been the lack of ideal reporters to identify T4SS translocated effectors and study T4SS function. Most reporter systems make use of fusions to reporter proteins, in particular, β-lactamase (TEM) and calmodulin-dependent adenylate cyclase (CYA), that allow detection of translocated enzymatic activity inside host cells. However, both systems require costly reagents and use complex, multistep procedures for loading host cells with substrate (TEM) or for analysis (CYA). Therefore, we have developed and characterized a novel reporter system using nanoluciferase (NLuc) fusions to address these limitations. Serendipitously, we discovered that Nluc itself is efficiently translocated by Legionella pneumophila T4SS in an IcmSW chaperone-dependent manner via an N-terminal translocation signal. Extensive mutagenesis in the NLuc N terminus suggested the importance of an α-helical domain spanning D5 to V9, as mutations predicted to disrupt this structure, with one exception, were translocation defective. Notably, NLuc was capable of translocating several proteins examined when fused to the N or C terminus, while maintaining robust luciferase activity. In particular, it delivered the split GFP11 fragment into J774 macrophages transfected with GFPopt, thereby resulting in *in vivo* assembly of superfolder green fluorescent protein (GFP). This provided a bifunctional assay in which translocation could be assayed by fluorescence microplate, confocal microscopy, and/or luciferase assays. We further identified an optimal NLuc substrate which allowed a robust, inexpensive, one-step, high-throughput screening assay to identify T4SS translocation substrates and inhibitors. Taken together, these results indicate that NLuc provides both new insight into and also tools for studying T4SS biology.

**IMPORTANCE** Type IV secretion systems (T4SS) are used by Gram-negative pathogens to coopt host cell function. However, the translocation signals recognized by T4SS are not fully explained by primary amino acid sequence, suggesting yet-to-be-defined contributions of secondary and tertiary structure. Here, we unexpectedly identified nanoluciferase (NLuc) as an efficient IcmSW-dependent translocated T4SS substrate, and we provide extensive mutagenesis data suggesting that the first N-terminal, alpha-helix domain is a critical translocation recognition motif. Notably, most existing reporter systems for studying translocated proteins make use of fusions to reporters to permit detection of translocated enzymatic activity inside the host cell. However, existing systems require extremely costly substrates, complex technical procedures to isolate eukaryotic cytoplasm for analysis, and/or are insensitive. Importantly, we found that NLuc provides a powerful, cost-effective new tool to address these limitations and facilitate high-throughput exploration of secretion system biology.

## INTRODUCTION

Bacterial type IV secretion systems (T4SS) are macromolecule delivery machines consisting of multisubunits that span the inner and outer cell membranes of Gram-negative pathogens. They are closely related to bacterial conjugation machines (1, 2). A number of Gram-negative bacterial pathogens use T4SS to transfer protein and/or DNA into eukaryotic cells, where these effectors then facilitate intracellular or extracellular bacterial replication through modulation of phagosome maturation and/or the immune response (1, 3, 4). T4SS have defining roles in infections caused by pathogens such as *Legionellales*, *Bartonella* spp., Brucella spp., Bordetella pertussis, Helicobacter pylori, *Anaplasmataceae*, and Rhizobium radiobacter (4–10).

To investigate the translocation of specific bacterial effectors into host cells, a variety of reporter systems have been developed. Fusions of bacterial effectors with adenylate cyclase (CYA) or β-lactamase (TEM-1) reporters have been the two mainstays used in investigation of type III, IV, and VI secretion systems (11–13). In particular, calmodulin-dependent CYA has proven sensitive (14, 15) and specific for detecting translocation, with the high specificity based on CYA only demonstrating enzymatic activity after combining with calmodulin in the host cell cytoplasm. However, these systems have several liabilities: for CYA, the time-consuming steps needed to extract intracellular cAMP for analysis by immunoassay and, for TEM-1, the high cost of the CCF-2 or CCF-4/AM fluorescent substrates and the complexity of substrate preloading into host cells.

We therefore set out to develop a next-generation reporter system to address these deficiencies. Nanoluciferase (NLuc) is a small 171-amino-acid monomer, which was coevolved from the 19-kDa OLUC-19 luminescent protein (16) from the deep sea shrimp (*Oplophorus gracilirostris*), along with a derivatized coelenterazine substrate to optimize luminescence output (17). Notably, it has enhanced structural stability and provides an over-150-fold-higher signal than the much larger firefly luciferase widely used in bioimaging and bioreporter studies (18–20). Furthermore, the furimazine substrate optimized for NLuc has low autoluminescence, enhancing the signal-to-noise ratio for this system. Based on these attributes, we hypothesized that NLuc might prove an optimal translocation reporter for T4SS. We therefore characterized use of NLuc as a T4SS translocation reporter and further investigated a highly efficient translocation signal unexpectedly identified in the N terminus of NLuc itself.

## RESULTS

### NLuc as a T4SS translocation reporter.

To investigate the potential of NLuc as a translocation reporter, we first constructed a fusion with RalF, a well-known T4SS translocated effector from Legionella pneumophila (13). In this construct, the RalF protein was fused to the C terminus of NLuc to preserve RalF’s C-terminal translocation signal (13). A 3×FLAG epitope was also added to the N terminus of the fusion protein to facilitate Western blot detection.

J774A.1 macrophages were then infected with either the T4SS-competent L. pneumophila strain, Lp02 *flaA* (dot/icm^+^), or T4SS-defective strain, Lp03 flaA (*dotA* mutant) (21), expressing the fusion protein. At 6 h postinfection (hpi), prior to significant bacterial replication and potential phagosomal degradation of the T4SS-defective *dotA* mutant strain, translocation was measured in saponin extracts of infected cultures by Western blotting (see Fig. S1A in the supplemental material). Of note, saponin, a cholesterol-dependent detergent, is used to selectively liberate eukaryotic cytoplasmic contents without solubilizing bacteria such as L. pneumophila, which do not contain cholesterol in their cell membranes. Therefore, in saponin extracts of eukaryotic cells, translocated protein is collected in the extract supernatant, while intact bacteria are pelleted and can be separately assayed as a control for protein expression levels in infecting bacteria.

We found that FLAG-tagged protein was only detected in eukaryotic cytoplasmic extracts during dot/icm^+^ infection, but not during *dotA* mutant infection, in rough proportion to the multiplicity of infection (MOI), supporting translocation of the NLuc:RalF fusion protein (Fig. S1A). Importantly, similar levels of FLAG epitope signal were detected in extract pellets from dot/icm^+^ and *dotA* mutant strains, indicating that differences in protein expression in these strains could not account for pronounced differences in protein detected in saponin extracts. Taken together, these results indicated that the NLuc protein could be transported by L. pneumophila T4SS when fused to RalF.

We then examined whether NLuc would retain its luciferase activity in a translocated fusion protein. Indeed, in eukaryotic cytoplasmic extracts, a robust, T4SS-dependent luminescence signal was observed on addition of furimazine substrate (Fig. S1B). Signal was approximately 100-fold higher in the wild type than *dotA* mutant T4SS mutant background and correlated with multiplicity of infection (Fig. S1B). In contrast, similar levels of NLuc activity were observed in extract pellets from dot/icm^+^ and *dotA* mutant infection (data not shown). These results suggested that the NLuc fusion protein was translocated into the macrophage cytoplasm in a manner that also preserved its luciferase activity.

### NLuc contains an intrinsic T4SS translocation signal.

Previously, the 20-amino-acid C terminus of L. pneumophila RalF effector was found to be both necessary and sufficient for translocation (13). However, translocation mediated by this C terminus alone occurred with reduced efficiency. Based on these prior observations, we tested whether the C terminus of RalF was similarly necessary and sufficient for NLuc translocation (Fig. S1C and D). Unexpectedly, essentially identical T4SS-dependent NLuc signal was observed in eukaryotic cytoplasmic extracts 6 h postinfection with L. pneumophila expressing NLuc fused with either the C-terminal 20 amino acids of RalF (NLuc:pRalF) or full-length RalF (NLuc:RalF). This result suggested that NLuc either compensates for the lower translocation efficiency previously associated with the C terminus of RalF or, alternatively, provides its own translocation signal.

**FIG 1:**
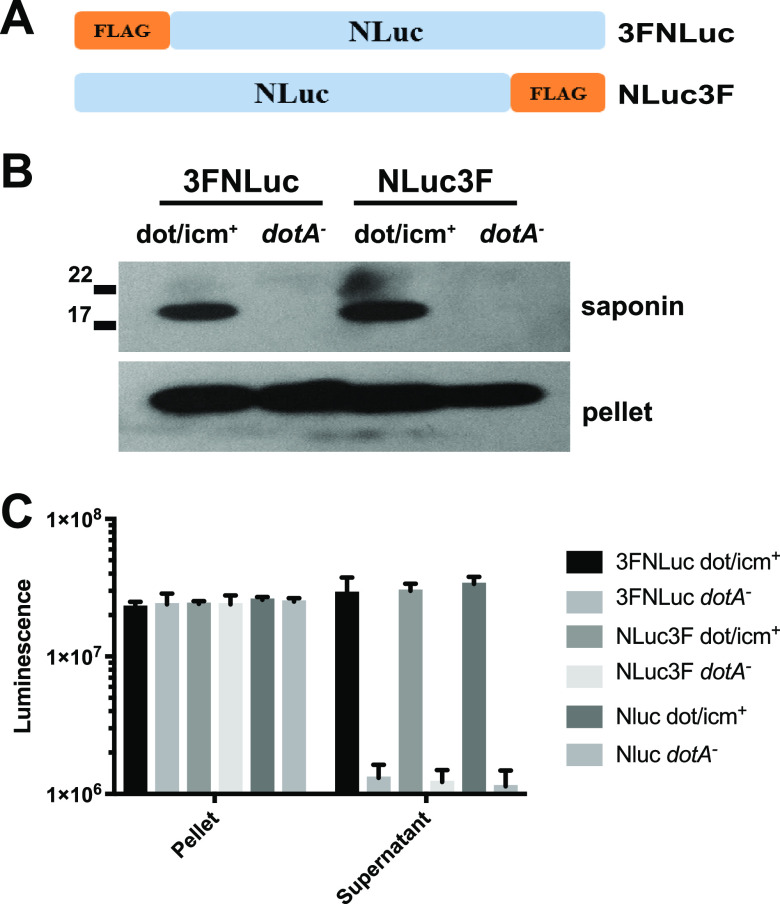
Intrinsic translocation signal in NLuc. (A) Diagram of NLuc constructs containing a 3×FLAG tag in the N terminus (3FNLuc) or C terminus (NLuc3F). (B) Western blot detection of translocation of 3FNLuc and NLuc3F expressed by T4SS-competent (Lp02, dot/icm^+^) or T4SS-incompetent (Lp03, *dotA* mutant) strains 6 h postinfection detected using anti-FLAG antibody. (C) Luminescence of translocated NLuc extracted from eukaryotic cytoplasm (supernatant) and insoluble pellet 6 h postinfection of J774A.1 cells infected with dot/icm^+^ or *dotA* mutant strains expressing NLuc with an N-terminal (3FNluc) or C-terminal (NLuc3F) 3×FLAG tag. Translocation of NLuc was evident and was not blocked by the 3×FLAG tag in either an N-terminal or C-terminal position. Plotted are the means and standard deviations (SD) of measurements from at least three biological replicates.

To investigate the possibility of an intrinsic translocation signal in NLuc, we expressed NLuc with either an N- or C-terminal 3×FLAG tag (Fig. 1A). At 6 hpi, we detected essentially identical T4SS-dependent translocation of these constructs by Western blotting (Fig. 1B). Similar results were observed for luciferase activity (Fig. 1C). NLuc was translocated just as efficiently in the absence of an N- or C-terminal 3×FLAG epitope based on a nanoluciferase assay, suggesting that the 3×FLAG itself neither contributed to nor interfered with the translocation phenotype, irrespective of location (Fig. 1C). Taken together, these results indicated the presence of an intrinsic T4SS translocation signal in NLuc.

**FIG 2:**
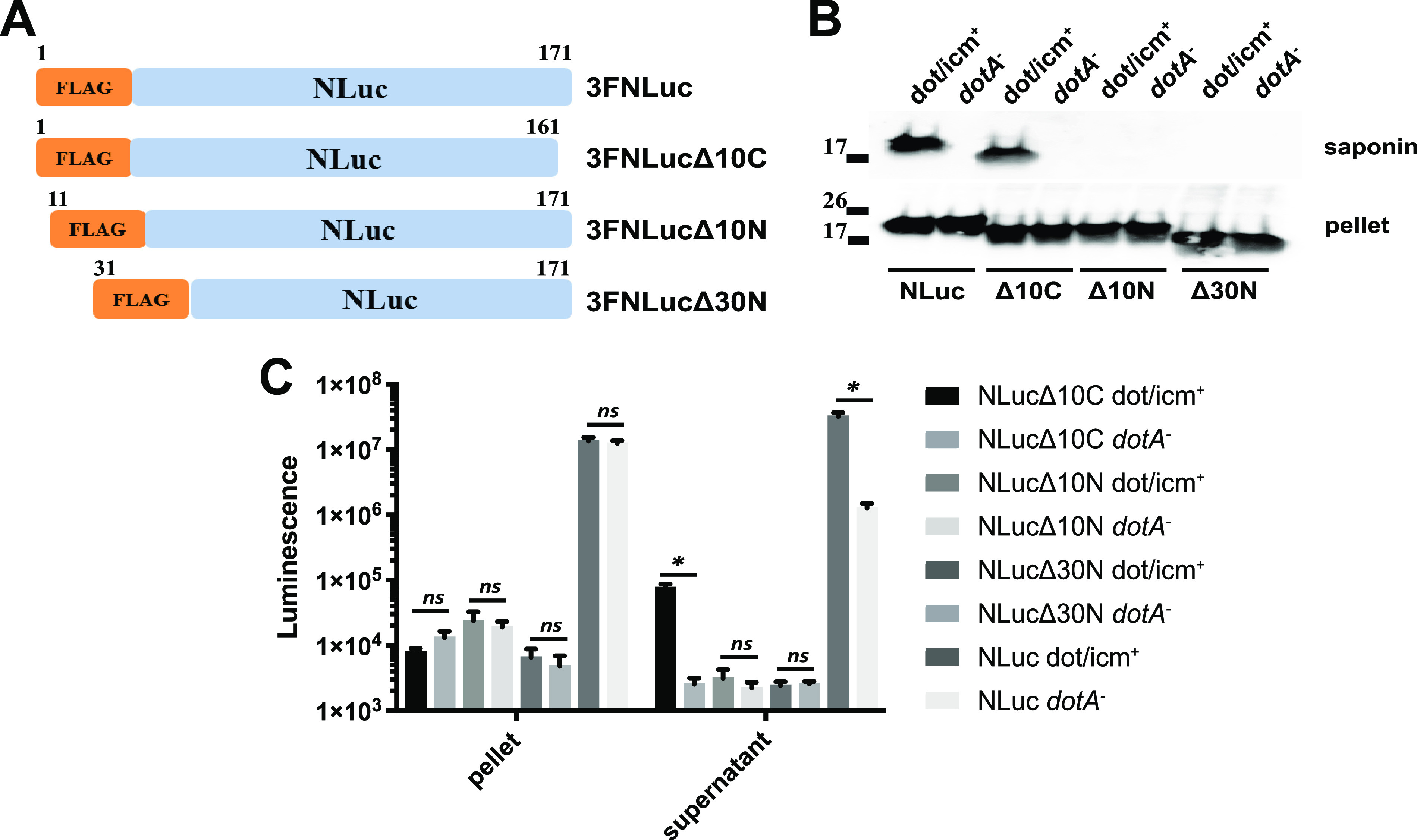
NLuc has an N-terminal translocation signal. (A) Illustration of NLuc deletion constructs used to establish location of an intrinsic translocation signal. Shown are wild-type 3FNLuc, 3FNLucΔ10C (with a deletion of 10 C-terminal amino acids), 3FNlucΔ10N (with a deletion of 10 N-terminal amino acids), and 3FNlucΔ30N (with a deletion of 30 N-terminal terminal amino acids). (B) Translocation of parental NLuc, NLucΔ10C, NLucΔ10N, and NLucΔ30N expressed by dot/icm^+^ or *dotA* mutant strains 6 h postinfection detected using anti-FLAG antibody. (C) Detection of translocated luciferase signal in extract supernatant and pellets from J774A.1 cells infected for 6 h with dot/icm^+^ and *dotA* mutant strains expressing C- and N-terminal NLuc truncations. Means and SD from three independent experiments are shown. Statistical comparisons between dot/icm^+^ and *dotA* mutant supernatant extracts used the Brown-Forsythe and Welch one-way analysis of variance (ANOVA) with Dunnett's multiple comparison *post hoc* test. *, *P* < 0.05; ns, no significant difference.

To identify the T4SS translocation signal in NLuc, we created a series of C- and N-terminal deletions of 3×FLAG-NLuc (Fig. 2A) and assayed for translocation 6 h postinfection. Deletion of the 10 C-terminal amino acids was associated with preserved T4SS-dependent protein translocation, as assessed by Western blotting (Fig. 2B), and a preserved ratio of dot/icm^+^ to *dotA* mutant luciferase signal (Fig. 2C). In contrast, deletion of either the 10 or 30 N-terminal amino acids abolished translocation. These data were consistent with a T4SS translocation signal residing in the N terminus of NLuc. Notably, although protein expression appeared relatively unaffected as assessed by Western blotting, the luciferase activity observed in bacterial pellets of all deletion constructs was substantially reduced, suggesting that both the N and C termini of NLuc are required for efficient luciferase activity.

### Characterization of the N-terminal translocation signal.

We next asked whether the N-terminal 30 amino acids of NLuc (partial NLuc [pNLuc]) were necessary and sufficient for T4SS-dependent translocation. To do so, we constructed fusions of TetR and tobacco etch virus (TEV) protease proteins fused to pNLuc with an N- or C-terminal 3×FLAG tag (Fig. S2A). Importantly, neither TetR nor TEV protease tagged with an N-terminal 3×FLAG alone were translocated by themselves (Fig. S2B). However, both proteins were translocated in a T4SS-dependent manner when fused with N- or C-terminal pNLuc (Fig. S2C and D). Interestingly, pNLuc:TetR was translocated more efficiently than TetR:pNLuc, while TEV:pNLuc was translocated slightly more efficiently than pNLuc:TEV. These results suggested that the specific fusion protein partner and its location relative to NLuc can influence either the availability of the translocation signal and/or efficiency of translocation.

### Dependence of NLuc translocation on IcmSW components of the type IV coupling transport complex.

**FIG 3:**
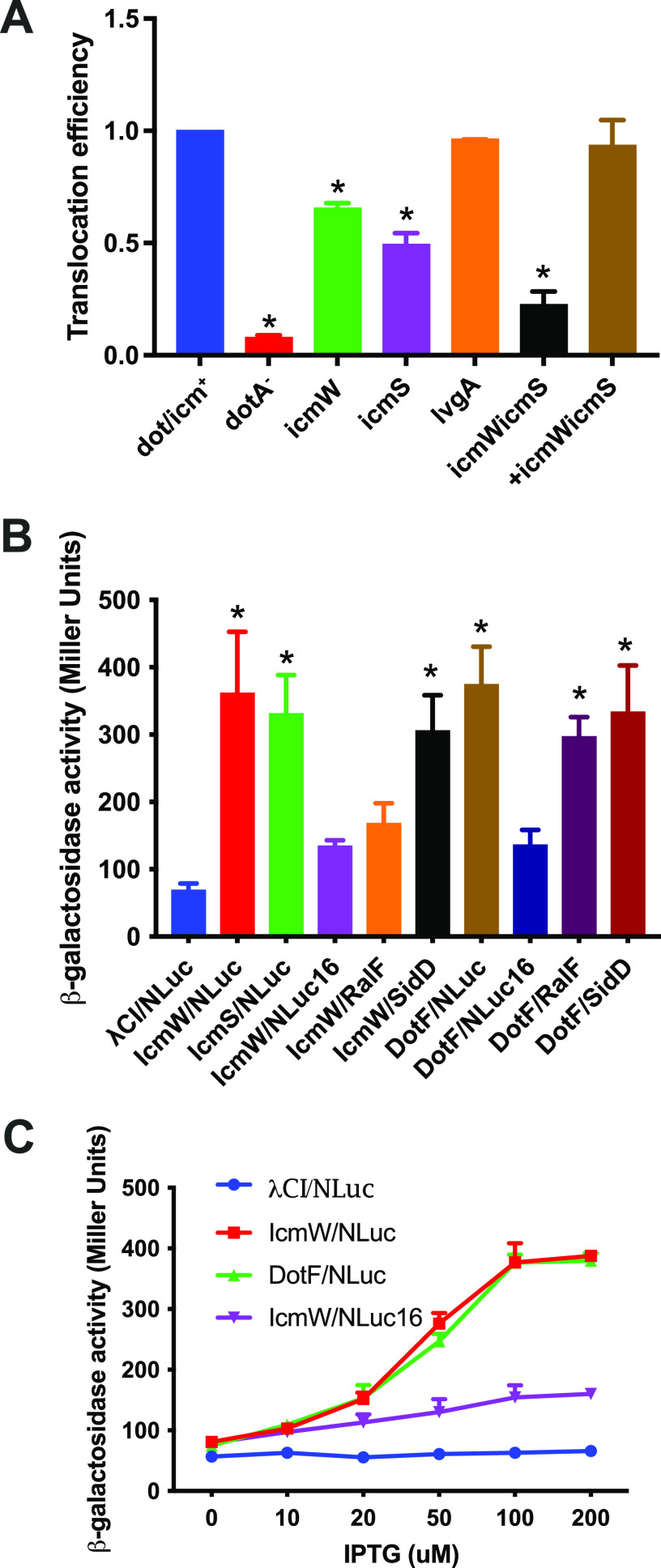
Dependence of NLuc translocation signal on IcmSW. (A) Luminescence-based translocation efficiency of NLuc in L. pneumophila dot/icm^+^, Δ*dotA*, Δ*icmW*, Δ*icmS*, Δ*lvgA*, Δ*icmSW*, and Δ*icmSW* complemented with plasmid-expressed *icmSW*. Extract supernatants and pellets were obtained 6 h postinfection at an MOI of 10. Translocation efficiency in mutants was normalized to signal from the dot/icm^+^ strain. Shown are the means and SD from two independent experiments. A one-way ANOVA with Dunnett's *post hoc* test was used for pairwise comparison with dot/icm^+^ control. *, *P* < 0.05. (B) BACTH assays to detect interactions of NLuc, RalF, and SidD prey with IcmW, IcmS, and DotF bait. Shown are means and SD of β-galactosidase activity from three independent experiments following induction of bait and prey fusion constructs with 100 μM IPTG. Statistical significance was determined using a one-way ANOVA test with Dunnett's multicomparison *post hoc* test. *, adjusted *P* < 0.05 for the comparison of the indicated condition with the λcI:β-flap/Nluc:αNTD expression vector combination (abbreviated λcI/NLluc) negative control. (C) Interaction of NLuc or NLuc16 with IcmW, DotF, or β-flap negative control. The means and standard deviations of β-galactosidase activity for two independent experiments are plotted versus IPTG concentration.

The chaperones IcmS, IcmW, and LvgA facilitate translocation of L. pneumophila effectors that lack canonical C-terminal E-block motifs (described further below) through participating in substrate recognition in the type IV coupling transport complex (22–24). We therefore examined their contribution to NLuc translocation through use of genetic knockouts. Interestingly, translocation was partially reduced in both isogenic Lp02 *icmW* and *icmS* mutants, but not in the Lp02 *lvgA* mutant strains (Fig. 3A). In the *icmSW* double knockout, translocation efficiency was reduced by ~84%, a defect that was fully complemented by an *icmSW* expression plasmid. The magnitude of *icmSW* translocation dependence was consistent with previous observations for translocation substrates that specifically bind to the IcmSW chaperone complex (25, 26).

Therefore, we next considered whether NLuc and its N terminus might directly interact with IcmSW and other protein components of the T4SS apparatus, as described for other effectors. To do so, we made use of a previously described bacterial two-hybrid system to test for such protein-protein interactions (27, 28). In this system, interaction of one potential protein partner, fused to λ *c*I, with a second protein fused to the N-terminal domain (NTD) of the α-subunit of RNA polymerase, leads to transactivation of a β-galactosidase reporter. Using this method, we detected strong interactions between NLuc:αΝΤD and λ *c*I:IcmW and between NLuc:αΝΤD and λ *c*I:DotF (Fig. 3B). Readout was dependent on isopropyl-β-d-thiogalactopyranoside (IPTG) concentrations used for induction of protein partners, supporting the specificity of our findings (Fig. 3C). Importantly, a negative control for baseline expression was established by testing coexpression of presumptively noninteracting λCI fused to the β-flap region of the RNA polymerase β-subunit and Nluc:αΝΤD (Fig. 3B, λ *c*I/NLuc), resulting in a comparatively low baseline transactivation that was not similarly induced by IPTG (Fig. 3C). As positive controls, we also examined interactions described previously (26, 29–32), detecting strong interactions (transactivation) between RalF and DotF, SidD and DotF, and SidD and IcmW. In contrast, as expected, we only detected a weak, not statistically significant interaction between the IcmSW-independent effector RalF and IcmW (Fig. 3B).

Importantly, when testing fusions with a version of NLuc with an N-terminal 15-amino-acid truncation (NLuc16:αNTD), we only observed weak, nonsignificant interactions with λ *c*I:IcmW and λ *c*I:dotF (Fig. 3B and C). These results suggested that the N-terminal translocation domain of NLuc contributed to interactions with the T4SS machinery components examined.

### Definition of translocation signal through random mutagenesis and targeted interruption of predicted secondary structure.

Based on the preceding results, we examined the 20 N-terminal amino acids for motifs previously associated with translocation. Previous studies identified a so-called E-block within the C-terminal T4SSb-dependent translocation signals in L. pneumophila (13, 33–35). This negatively charged motif, defined by enrichment in glutamate and aspartate residues (35), has been proposed to interact with cytoplasmic-facing, positively charged amino acids of the DotM, T4SS protein to initiate translocation (22).

**FIG 4:**
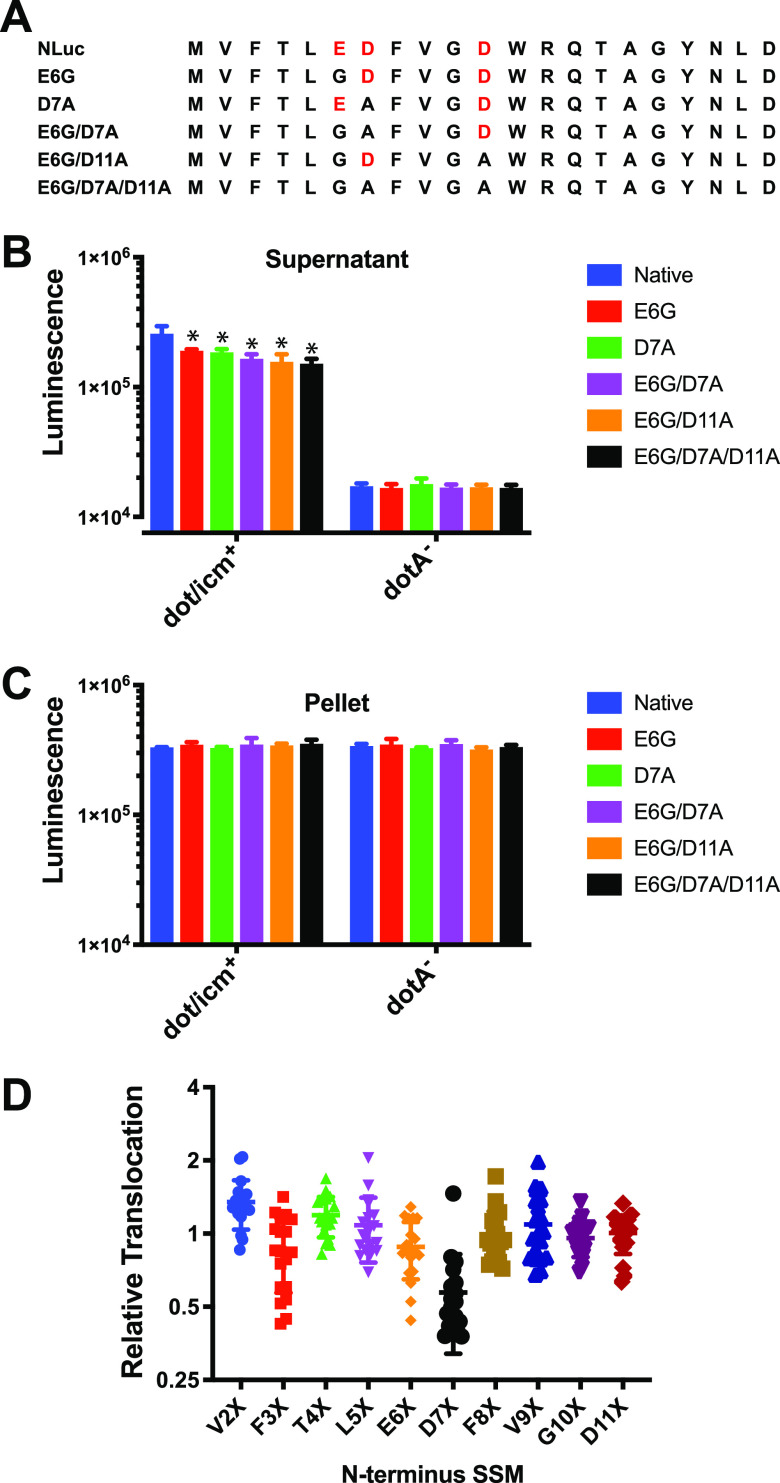
The N-terminal translocation signal does not substantially depend either the presence of negatively charged amino acids or its primary amino acid sequence. (A) Negatively charged amino acids (red type) in the N terminus of NLuc were mutated to neutral alanine or glycine. J774A.1 cells were infected for 6 h at an MOI of 10 with L. pneumophila strains expressing NLuc with the indicated mutations. (B and C) Saponin extract supernatant (B) and pellet (C) were then assayed for nanoluciferase activity. Data shown are the means and SD of three independent experiments. Statistical comparisons between parental NLuc and each mutated NLuc were determined by ANOVA with Dunnett’s *post hoc* test. *, *P < *0.05. (D) Scanning, random mutagenesis of V2-D11 amino acids in NLuc. Each data point represents an NLuc variant with a unique amino acid substitution at the indicated position. Relative translocation was calculated based on the ratio of luciferase activity in the saponin extract to that of the bacterial pellet at 6 h postinfection with the *dot/icm*^+^ strain expressing the indicated mutant at an MOI of 10. Values were normalized to translocation of wild-type NLuc and are the average translocation values from two independent experiments.

The presence of aspartic and glutamic acid residues in the N terminus of NLuc (Fig. 3A and Fig. S3A) suggested potentially similar functionality. To characterize their contribution to translocation, we replaced these negatively charged amino acids sequentially with charge-neutral, nonpolar alanine and/or glycine residues (Fig. 4A). At 6 hpi, T4SS-dependent translocation of these NLuc mutants was measured (Fig. 4B) based on luminescence signal, and it was noted to be reduced in a modest, stepwise fashion from ~28% to ~44% with single (a Glu-to-Gly change at position 6 [E6G] or D7A), double (E6G/D7A or E6G/D11A), or triple mutations (E6G, D7A, and D11A). Importantly, intrinsic luciferase activity of NLuc mutants was no different from that of the wild-type NLuc (Fig. 4C, bacterial pellets), suggesting that lower luciferase activity in eukaryotic cytoplasmic extracts resulted from reduced translocation. Based on a partial phenotype, we inferred that other features in the N terminus of NLuc must also contribute significantly to its translocation.

To investigate more broadly the potential involvement of specific amino acid sequences in the N-terminal translocation signal, we replaced each amino acid from V2 to D11 sequentially through randomization of each respective codon. We then compared the translocation efficiencies normalized to expression in bacterial pellets (to control for effects of mutations on intrinsic luciferase activity) during J774A.1 cell infection. Surprisingly, all mutations were well tolerated, except positions 3F, 6E, and 7D, where reduced translocation of up to 70% was observed (Fig. 4D, Table S1). However, no single substitution was able to fully block translocation. Mutations either variably decreased, had no effect, or in some cases increased absolute luminescence (Table S1).

**FIG 5:**
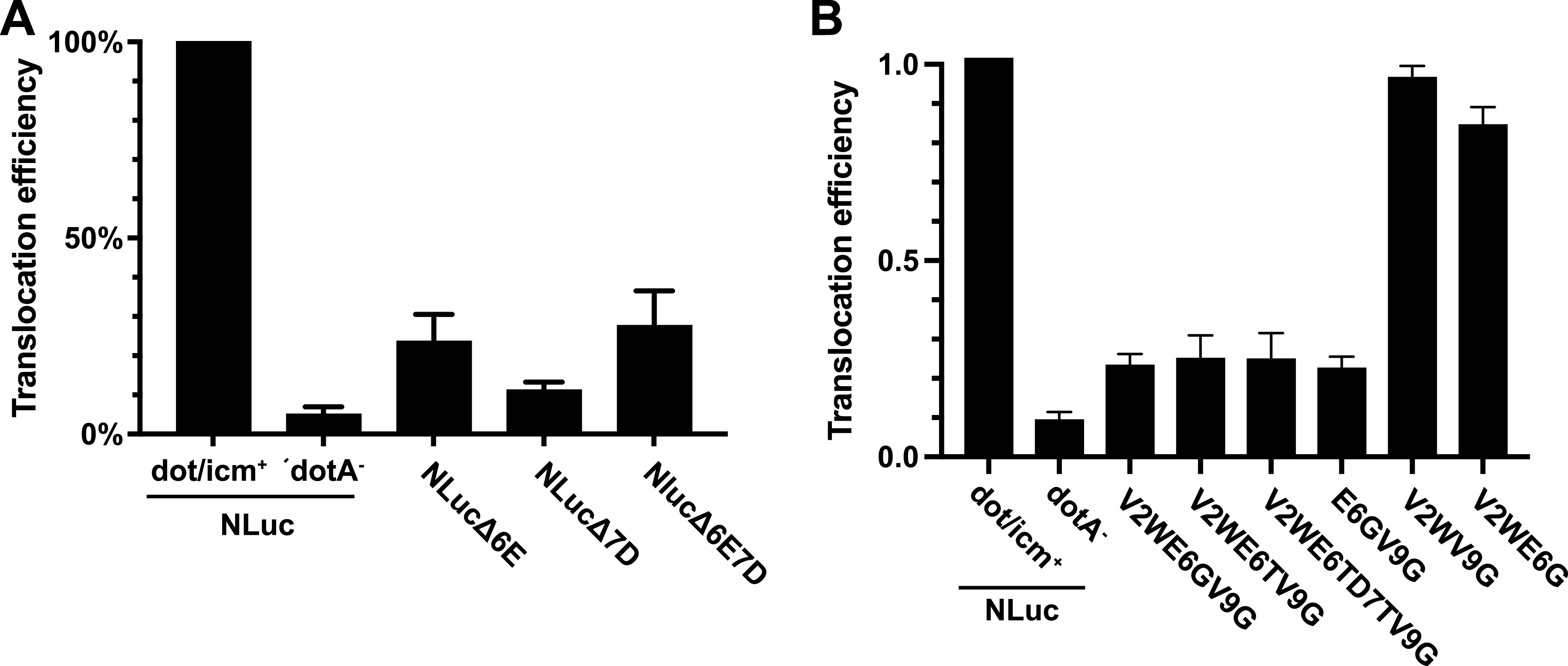
Predicted disruption of the α-helical region in the N terminus of Nluc abrogates translocation. (A) Translocation efficiency of parental NLuc in the dot/icm^+^ or dotA mutant strains and NLucΔ6E, NLucΔ7D, and NLucΔ6EΔ7D expressed in the dot/icm^+^ strain. Translocation efficiencies of mutants are the ratios of luciferase activity in the saponin extract supernatant to that of the bacterial pellet normalized to the translocation efficiency of wild-type NLuc, performed at 6 h postinfection of J774A.1 cells at an MOI of 10. Bars are the means and SD from two independent experiments. (B) Translocation efficiency of wild-type NLuc and the indicated multisite mutants, expressed in the dot/icm^+^ strain. Bars shown are the means and SD from three independent experiments.

Due to only partial abrogation of translocation by single amino acid mutations, we considered whether secondary structure might contribute to NLuc recognition by T4SS. The RaptorX convolutional neural network-based, secondary structure prediction program (36) predicted an α-helical region in the N terminus of NLuc corresponding to the surface-exposed α-helix observed in the crystal structure of NLuc in PDB 5IBO (Table S2) (37). Single amino acid substitutions such as E6G, D7A, D11A, or their combinations did not disrupt the predicted α-helical structure. However, Δ6E, Δ7D, and Δ6E7D deletions did so (Table S2) and experimentally were correspondingly noted to decrease normalized translocation up to 94% for Δ7D, with subtraction of background signal from the *dotA*-T4SS-incompetent control (Fig. 5A). A similar elimination of α-helical structure was predicted by AlphaFold2 (Fig. S3) (38). Interestingly, all three deletions increased luminescence signal roughly 4- to 6-fold in pellets (Table S1), suggesting opposite effects on enzymatic and translocation activities.

Based on these data, we designed degenerate primers to randomly mutagenize positions V2 through W12. Over 86% of the ~7,000 clones analyzed showed >10-fold decreased luminescence and were not further assessed (Fig. S4). We selected ~30 NLuc mutants showing high luciferase activity and evaluated translocation efficiency (Table S2). Interestingly, these highly active luciferase variants showed changes in only one to four amino acid positions compared with the native nanoluciferase sequence, suggesting relatively stringent conservation requirements in this region for optimal luciferase activity. Based on observed mutations, we also constructed V2WE6G and E6GV9G. Among all variants examined, V2WE6TV9G, V2WE6TD7TV9G, V2WE6GV9G, and E6GV9G showed ~5-fold decreased translocation compared to parental NLuc (Fig. 5B) However, translocation of V2WV9G and V2WE6G were unaffected (0.97 translocation efficiency). Notably, all of the former, and V2WV9G, showed a predicted disruption of α-helical structure based on RaptorX (Table S2), while none showed disruption based on AlphaFold2 (data not shown). Taken together, our results suggested that the surface-exposed, N-terminal α-helical secondary structure may be a critical component of the *icmSW*-dependent T4SS signal.

### Developing a fluorescent T4SS translocation reporter using split, superfolder GFP.

Based on its native T4SS signal, we next considered whether it would be possible to tether the Nluc protein to a split-GFP superfolder (sfGFP) fluorescent reporter (39) to provide a specific and temporal readout of T4SS translocation without the need for extraction of eukaryotic cytoplasm. The utility of split-sfGFP is predicated on *in vivo* self-assembly of the individually nonfluorescent, 16-amino-acid GFP11 fragment and complementary GFP1-10 OPT (GFPopt) domains with resulting restoration of GFP fluorescence (39, 40). We constructed a split-sfGFP reporter system on this basis.

We initially checked *in vivo* assembly of GFP11 fused with either the positive translocation signal control, pRalF, or NLuc (GFP11:pRalF and NLuc:GFP11, respectively), and GFPopt in Escherichia coli (Fig. S5A). Coexpression of either of the two GFP11 fusions with GFPopt resulted in increased fluorescent signal (*P < *0.05), i.e., significantly above the background signal represented by expression of GFPopt alone. These results indicated *in vivo* reconstitution of functional sfGFP in E. coli when GFP11 was available as a fusion protein with either pRalF or NLuc translocation signals.

**FIG 6:**
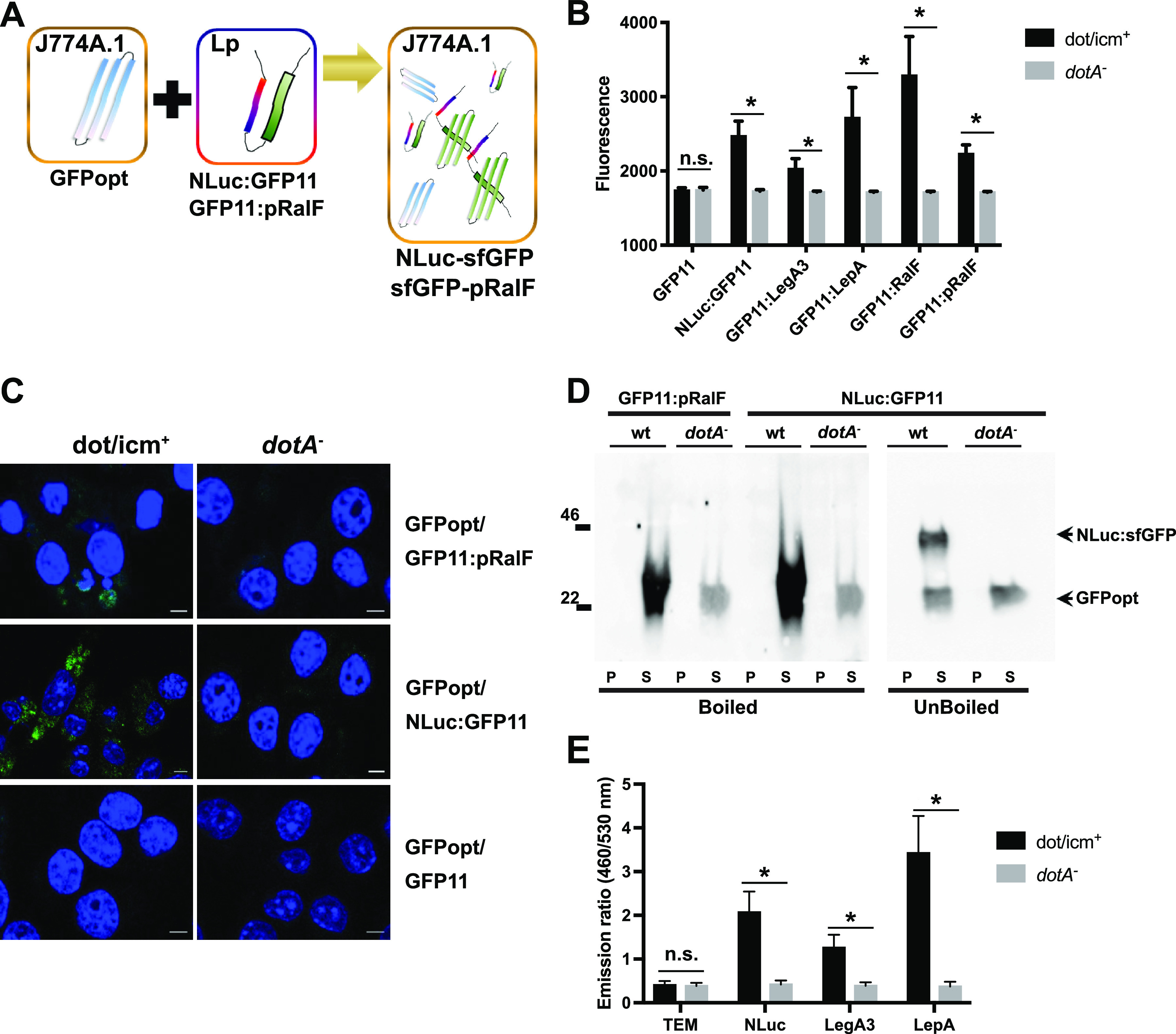
Combining superfolder GFP and NLuc systems to build a bifunctional, internally orthogonal T4SS-dependent high-throughput translocation assay. (A) Nonfluorescent GFPopt is expressed constitutively in J774A.1 macrophages. NLuc:GFP11 or GFP11:pRalF translocated into infected macrophages from L. pneumophila combines with GFPopt to form fluorescent sfGFP detectable in microplate assay or by confocal microscopy. Translocated luciferase activity can be detected separately as an orthogonal measure after extraction of eukaryotic cytoplasm or in a one-step assay. (B) In the experiment shown, J774A.1 GFPopt macrophages, seeded in 96-well plates at 1 × 10^5^ cells per well, were infected for 24 h with L. pneumophila expressing either NLuc-GFP11, GFP11:LegA3, GFP11:LepA, GFP11:RalF, or GFP11:pRalF. Assembly of sfGFP was quantified by fluorescent signal. Data shown are the means ± SD from two biological replicates with five or six technical replicates per experiment. (C) Alternatively, assembly of fluorescent sfGFP (green) was detected by confocal microscopy with DAPI counterstain (blue). Scale bars, 5 μm. (D) Cytoplasmic extracts and pellets of J774A.1 GFPopt cells infected for 24 h with wild-type (wt) dot/icm^+^ or *dotA* mutant strains expressing GFP11:pRalF or Nluc:GFP11 were analyzed by Western blotting using polyclonal anti-GFP antibody. Samples from Nluc:GFP11 infections were loaded onto SDS-PAGE gels in standard SDS loading buffer with (boiled) or without (unboiled) prior incubation at 95°C for 5 min. The ~46-kDa band observed in the unboiled dot/icm^+^ supernatant sample is the size predicted for an assembled complex of Nluc:GFP11 (22 kDa) and GFPopt (24 kDa). (E) TEM-1 β-lactamase-based translocation assay. J774A.1 cells were infected with dot/icm^+^ or *dotA* mutant strains carrying plasmids encoding NLuc-TEM, TEM-LegA3, TEM-LepA, or TEM alone (negative control). T4SS-mediated translocation of the fusion proteins into host cells was assessed by measuring CCF4-AM hydrolysis 4 h postinfection reflected in the ratio of fluorescent signal at 460 nm (cleaved substrate) to 530 nm (uncleaved substrate). Data shown are the means ± SD from two biological replicates with three or four technical replicates per experiment. *, *P < *0.05, two-way ANOVA with Sidák *post hoc* test for comparisons of translocation in dot/icm^+^ versus *dotA* mutant strains. n.s., not significant.

Based on these data, J774A.1 host cells expressing GFPopt were infected with L. pneumophila strains expressing either NLuc:GFP11, GFP11:LegA3, GFP11:LepA, GFP11:RalF, or GFP11:pRalF in 96-well microplate assays (Fig. 6A). At 24 h postinfection, we observed a significant (Fig. 6B) T4SS-dependent GFP signal for strains expressing either NLuc:GFP11 or *L pneumophila* effector fusion proteins. As expected, control strains expressing GFP11 in the absence of a translocation signal did not show dot/icm^+^-dependent signal. Signal for dot/icm^+^-expressing NLuc:GFP11 was 40% greater than dot/icm^+^-expressing GFP11:pRalF, suggesting that NLuc either provided a stronger translocation signal for the GFP11 reporter fragment and/or allowed for more efficient or effective sfGFP reconstitution. Comparisons between signal from dot/icm^+^ and *dotA* mutant infection yielded a Z′ of 0.32 ± 0.01 for Nluc:GFP11 and −0.11 ± 0.28 for GFP11:pRalF, respectively (Fig. S5B), suggesting that the former, but not the latter, had robustness compatible with use in high-throughput translocation inhibition assays. Presumably, there could be additional improvement in Z′ with further assay optimization. It is also possible that a stronger translocation signal, perhaps whole RalF in this assay, might also provide a statistically more powerful assay.

To investigate the utility of these reporters in image-based analysis, we examined infections with these constructs using confocal microscopy (Fig. 6C). Confocal images showed host cell cytoplasmic fluorescent signal during infection of J774A GFPopt cells with dot/icm^+^ but not with *dotA* mutant strains expressing either GFP11:pRalF or NLuc:GFP11 fusions. However, signal was not observed when the GFP11 fragment was expressed alone. The latter control, also shown in Fig. 6B, indicated that GFP signal did not result from leakage of GFP11 into the host cytoplasm as a result of bacterial lysis during the course of infection. Similar to microwell experiments, T4SS-dependent translocation signal was noticeably greater for NLuc::GFP11 than for GFP11::pRalF.

Western blot analysis provided additional indirect evidence for translocation-dependent assembly of sfGFP in the host cell cytoplasm (Fig. 6D). Notably, during infection with dot/icm^+^ strain expressing GFP11:pRalF, a very strong ~24-kDa band, the expected size of host cell-expressed GFPopt, was detected with polyclonal anti-GFP antibody by Western blotting after denaturing electrophoresis. In contrast, during infection with the *dotA* mutant strain, the 24-kDa band was significantly weaker, suggesting much smaller amounts of GFPopt protein. We therefore reasoned that T4SS-dependent translocation of GFP11:pRalF led to stabilization and higher accumulation of the 24-kDa GFPopt in the context of an assembled sfGFP complex, consistent with fluorescence microscopy results. Presumably, during denaturing conditions used for SDS-PAGE analysis, the larger sfGFP complex was dissociated, and the polyclonal anti-GFP antibody was also unable to detect the very small GFP11 portion of the translocated fusion protein.

Similar T4SS-dependent stabilization of the 24-kDa GFPopt band was observed during infection with L. pneumophila expressing NLuc:GFP11. However, if extracts were not boiled prior to gel loading, a strong ~46-kDa band was detected during infection with the T4SS-compentent dot/icm^+^ strain. This band was likely accounted for by formation of the complex between 22-kDa NLuc:GFP11 and 24-kDa GFPopt. Under these conditions, we infer that remaining weak 24-kDa band in extract supernatants observed during both dot/icm^+^ and *dotA* mutant infection represents GFPopt protein that did not participate in sfGFP complex formation.

To compare the strength of the NLuc translocation signal with other know T4SSb translocation signals, we fused NLuc to the N terminus of TEM-1 β-lactamase, and LegA3 (strong signal) and LepA (weak signal) with the C terminus of TEM-1 β-lactamase (Fig. 6E) (41, 42). Expression of the TEM-1 gene alone served as a negative control. Translocation efficiency was assessed by measuring the emission ratio (460 nm/530 nm) after loading macrophages with the CCF4-AM fluorescent β-lactamase substrate. Based on this assay, Nluc-driven translocation was as at an intermediate level between LegA3 and LepA translocation signals.

### Single-step, NLuc-based, high-throughput screening assay.

Furimazine, a coelenterazine analog, has been the substrate of choice for nanoluciferase-based applications (17–19). However, the high permeability of *Legionella* to furimazine necessitates time-consuming steps to separate eukaryotic cytoplasm from bacteria prior to nanoluciferase-based translocation assessment. We therefore considered whether identification of a less bacterial-penetrant, coelenterazine substrate might enable a simpler, one-step translocation assay.

The bacterial membrane permeability index of available coelenterazine analogs (Table S3) was empirically determined based on a ratio of luminescence from bacteria suspended in phosphate-buffered saline (PBS) to luminescence of bacteria permeabilized in radioimmunoprecipitation (RIPA) buffer containing 1% NP-40 and 1% sodium deoxycholate. Results were normalized to activity of purified nanoluciferase added to PBS and RIPA alone, as RIPA buffer variably inhibited or stimulated activity of nanoluciferase substrates up to 10-fold. Coelenterazine h, i, cp, and n showed the lowest permeability index (3- to 5-fold lower than furimazine) among nanoluciferase substrates with high signal.

**FIG 7:**
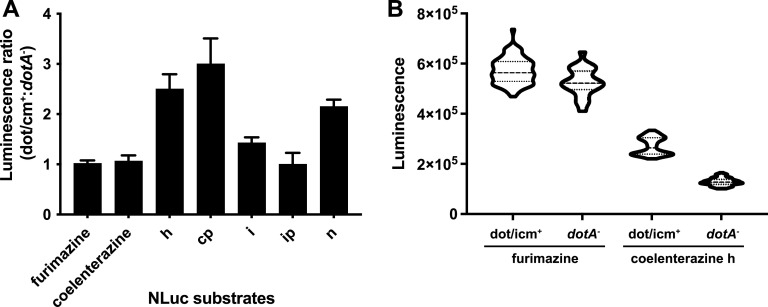
Identification of coelenterazine analogs compatible with a one-step NLuc translocation assay. (A) Ratio of luminescence in assay wells after a 6-h infection of macrophages with dot/icm^+^ versus *dotA* mutant strains expressing NLuc on addition of the indicated coelenterazine analogs (coelenterazine h, cp, i, ip, and n, labeled with their single-letter suffix) in saponin lysis buffer, i.e., procedurally a single-step translocation assay. Data show are means and SD for 10 determinations obtained during three independent experiments normalized to the furimazine control. (B) Luminescence from one-step translocation assays in high-throughput format during infection with dot/icm^+^ or*dotA* mutant strains expressing NLuc in the presence of furimazine or coelenterazine h substrates. Violin plots show combined results from two independent experiments, each with 40 technical replicates per variable, which were used to determine the Z′ described in the text. In one-step assays, clear separation between T4SS-competent (dot/icm^+^) and -incompetent (*dotA* mutant) signal was observed with the coelenterazine h substrate.

Based on these data, we then compared the ability of high-penetrant analogs (coelenterazine f and furimazine) and low-penetrant analogs to serve as discriminatory substrates in one-step, NLuc translocation assays in a 96-well microplate format. In these one-step assays, substrate was added directly to infected cells at 6 hpi in the presence of 0.2% saponin. Those with low bacterial permeability (h, cp, i, and n) displayed distinct and statistically significant *(P < *0.0001, Mann-Whitney U test) T4SS-dependent luminescent signal (Fig. 7A, Table S2). Coelenterazine h was then compared with furimazine tested in a one-step, 96-well high-throughput assay format, the former yielding a discriminatory Z' of 0.52 ± 0.05 and the latter a nondiscriminatory Z' of −5.9 ± 0.3 during comparisons of *dot/icm*^+^ and *dotA* mutant infection (Fig. 7B). Notably, NLuc:GFP11 performed similarly to NLuc in single-step nanoluciferase translocation assays with coelenterazine h substrate (Fig. S5C). This suggests the potential use of bifunctional NLuc:GFP11 in combination with GFPopt J774A.1 cells to provide orthogonal confirmatory readouts for T4SS translocation in the same high-throughput screening assay, providing a highly efficient system for studying biochemical or genetic perturbation of T4SS function.

## DISCUSSION

Our results identified nanoluciferase (NLuc) as a new reporter for analysis of T4SS-dependent translocation with several propitious properties. The small 17-kDa NLuc reporter provided robust translocation signal when fused to both N and C termini of proteins examined. It could be detected using a number of inexpensive nanoluciferase substrates or by Western blotting using epitope tags or nanoluciferase antibody and was compatible with high-throughput assay formats. It offered considerable advantages over other systems in current use, which require complex techniques for isolation or detection of reporter enzyme substrates at considerable expense.

As NLuc is an evolved, eukaryotic, deep sea shrimp luciferase, a surprising finding was the presence of a very strong N-terminal, T4SSa-dependent translocation in Nluc itself. Notably, the specific mutations introduced in development of NLuc (16, 17), T2V, A6E, and R12Q (NLuc numbering), from *Oplophorus* luciferase coincidentally appeared to enhance translocation efficiency based on our mutagenesis analysis (Table S1) and may have serendipitously contributed to the NLuc translocation signal.

The existence of an N-terminal T4SSb translocation signal in L. pneumophila to the best of our knowledge has not been previously established through deletion or mutagenesis experiments. However, of interest, 8 Leg proteins and LepA were previously found to be translocated either as fusions to the N terminus of CYA or to the C terminus of TEM β-lactamase (43–45). These results could be consistent with a C-terminal signal that remains active despite downstream fusion protein sequence (44, 45), or potentially, a signal emanating from other regions of these effectors, including the N terminus. Of note, many studies seeking to identify T4SSb translocated effectors have been based on screens in which candidate effector proteins were fused to the C terminus of reporters (46, 47), thereby potentially ignoring strong N-terminal signals. Internal recognition sites are also postulated for some IcmSW substrates (26), suggesting C-terminal signals are not obligatory. Therefore, the discovery of a strong N-terminal signal is not completely surprising.

Biological investigation suggested that NLuc translocation was IcmSW dependent, as ~90% of NLuc translocation was abrogated in an *icmSW* background. Furthermore, analysis using a bacterial two-hybrid system supported direct interaction of the NLuc with IcmSW T4SS chaperones. In particular, the interaction of the N terminus of NLuc with IcmW was also suggested by loss of interaction in N-terminal NLuc deletions. It is possible that IcmS and IcmW proteins contribute to the folding of NLuc into a conformation accessible to the type IV secretion apparatus, functions of these chaperones suggested previously (26), or recruit NLuc to the T4SS through their association with the type IV coupling protein complex (48).

In the bacterial two-hybrid system, we also noted interaction of NLuc and RalF with the inner membrane T4SS component DotF. Previously, a two-hybrid analysis by Luo and Isberg et al. (32) identified interactions of DotF (amino acids 28 to 123) with RalF, SidA to SidH, and SdeC using a two-hybrid system. Similarly, Sutherland et al. (29) found in two-hybrid assays that a similar region of DotF (amino acids 50 to 123), corresponding to DotF’s transmembrane and a portion of its periplasmic domain, but notably not its cytoplasmic domain, was required for the strong, two-hybrid signal. Importantly, they also found that DotF did not recognize the C-terminal translocation signals of either RalF or SidG. Therefore, those authors questioned the importance of DotF for T4SS substrate recognition, as DotF neither appeared to bind the canonical C-terminal T4SSb translocation signal nor was its cytoplasmic domain, presumably accessible for interaction with as-yet-untranslocated substrates, contributory.

Unfortunately, the N-terminal 200 amino acids of DotF were not resolved in recent cryo-electron microscopy (cryo-EM) structures of the Dot/Icm T4SS apparatus (PDB 7MUD) (49). Without structural information and additional experimentation, it is therefore at this time not possible to determine whether potential interactions of NLuc with extracytoplasmic domains of DotF may be biologically relevant. For example, it is possible that the interacting portion of DotF, amino acids 50 to 123, lies within the T4SS channel and might constitute one of many interactions contributing to secretion of a subset of T4SS substrates. In our study, the N-terminal secretion signal from NLuc was necessary for the two-hybrid interaction with DotF, although we did not assess the corresponding portion of RalF involved with this interaction. This leaves two possibilities: either the interactions with DotF are biologically relevant, or alternatively, the interaction of DotF with NLuc, as is potentially the case for other T4SS substrates, is an artifact based on altered conformations and binding affinities that may affect two-hybrid assays (29, 50, 51).

Notably, based on the crystal structure of NLuc, previously solved to 1.95 Å (37), its N terminus is predicted to be surface exposed, potentially allowing it to interact directly with T4SS translocation machinery. In particular, we noted enrichment in negatively charged amino acids in the N terminus, reminiscent of previously identified C-terminal translocation signals. However, specific replacement of these negatively charged amino acids either singly or in combination with uncharged amino acids or random scanning mutagenesis of each single N-terminal amino acid position had only partial or no effect on translocation, suggesting a potential secondary role of the primary amino acid sequence in the N-terminal secretion signal. In contrast, disruption of the existing α-helical secondary structure, as predicted by RaptorX, either through amino acid deletion or multiple amino substitutions, almost completely abolished translocation, with the single exception of V2WV9G. Overall (Table S2), 8 of 9 proteins with predicted α-helical disruption had severe defects in translocation, and the 33 proteins without alpha-helical disruptions had minor if any translocation defect (*P* < 0.0001, Fisher’s exact test, for an association of disruption of secondary structure with a translocation defect). AlphaFold2 also predicted abolition of α-helical secondary structure in amino acid deletion mutants associated with translocation defects; in contrast, it predicted preservation of α-helical structure in variants with multiple amino acid substitutions, including those defective in translocation. However, the ability of AlphaFold2 to accurately predict structural alterations associated with missense mutations has been questioned, based on weighting of its algorithm toward recapitulation of existing PDB structures (52). We acknowledge the limitations of secondary and tertiary structure prediction algorithms. Firm resolution would require X-ray or cryo-EM determinations for all structures, which is beyond the scope of the current study. Nevertheless, taking the mutagenesis data and structural predictions together, we suggest that surface-exposed α-helical secondary structure may be important for recognition by IcmSW-associated T4SS machinery.

We initially considered use of NLuc in one-step assays with a simple addition of luciferase substrate in the presence of saponin detergent, without need for extraction of eukaryotic cytoplasm and physical separation from infecting organisms. However, *Legionella* proved relatively permeable to commonly used furimazine substrate, perhaps related to *Legionella’s* overall increased permeability to small molecules (e.g., Gram-positive antibiotics) compared with *Enterobacterales* (53). Fortunately, less-permeable coelenterazine analogs were identified (Table S1) that proved compatible with use in one-step T4SS translocation assays in a high-throughput screening format. Furthermore, NLuc provided again an excellent fusion partner with GFP11, supporting its use as a translocation signal in an alternative, single-step translocation assay based on detection of sfGFP in host cells. Although sfGFP assays have been previously used in translocation assays (54–56), a particularly exciting aspect of our bifunctional NLuc-based sfGFP constructs for high-throughput screening drug discovery is that orthogonal confirmation of sfGFP results could be performed in the same screening wells using the alternative nanoluciferase readout.

Taken together, our data support use of NLuc-based T4SSb translocation assays. It is possible that NLuc may also prove a useful reporter for study of other secretion systems, potentially without the confounding NLuc translocation signal recognized by T4SSb, which would further extend its utility. NLuc in addition provides a system for further exploration of the nature of T4SS secretion signals, which likely include critical secondary structural motifs.

## MATERIALS AND METHODS

### Bacterial strains and plasmids.

Bacterial strains, plasmids, and eukaryotic cell lines used in this study are listed in Table S4 in the supplemental material. Escherichia coli NEB-5α (New England Biolabs, Beverley, MA) was grown in Luria broth (LB) medium (BD, NJ). Legionella pneumophila Lp02 *flaA* and Lp03 *flaA* strains were grown on buffered charcoal yeast extract (BCYE) medium supplemented with 100 μg/mL of thymidine (21).

### Cells and materials.

Murine J774A.1 (ATCC TIB-67, American Type Culture Collection, Manassas, VA) macrophage cell lines were cultivated in RPMI 1640 (Thermo Fisher Scientific, MA) with 9% heat-inactivated, iron-supplemented calf serum (GemCell, Gemini Bio-Products, CA) in a humidified 5% CO_2_ atmosphere at 37°C. Furimazine was from Promega (catalog number N-110; Madison, WI) or from AOBIOUS (Gloucester, MA) for bacterial permeability studies. Coelenterazine and coelenterazine f, h, cp, fcp, hcp, i, ip, and n were from Biotium (Fremont, CA), and coelenterazine e, e-f, v, and 400a were from Nanolight Technology (Pinetop, AZ). Coelenterazine analogs were dissolved at 250 μg/mL in ethanol. Prior to use, 1 μL of each substrate solution was added to 1 mL of PBS or RIPA lysis buffer (Cell Signaling Technology, Danvers, MA) for NLuc experiments. Furimazine (Promega) was used at a 1:1,000 dilution from purchased stock.

### Generation of bacterial strains expressing NLuc fusion proteins.

PCR products for gene or gene fragments were digested with appropriate restriction enzymes and cloned into similarly digested pXDC61 (EcoRI/KpnI) or pRetroX (BamHI/EcoRI). The NLuc sequence was amplified from E. coli strains harboring pNL1.1 (Promega, Madison, WI) (primers are listed in Table S5) and cloned into the vector pXDC61 (57), with or without incorporation of a 3×FLAG tag immediately after the start codon or before the stop codon. For the construction of NLuc:RalF fusion proteins, the whole gene or the terminal 20 amino acids of RalF (amino acids 355 to 374) was amplified from L. pneumophila and cloned into pXDC61-NLuc using restriction enzymes KpnI and BamHI. Fusions of partial NLuc and TEV or TetR were constructed from corresponding gBlocks (Integrated DNA Technologies, Coralville, IA) and cloned into pXDC61. GFP11 fused with partial RalF or NLuc was cloned into pXDC61 using its EcoRI and KpnI restriction sites. GFPopt (synthesized as a gBlock; Integrated DNA Technologies) was amplified with indicated primers and cloned into the pRetroX vector using restriction enzymes BamHI/EcoRI to create pRetroX-GFPopt. Plasmids were transformed into E. coli NEB-5α and/or L. pneumophila by electroporation as previously described (57) or from GFPopt transfected into J774A.1 cells as described below.

### NLuc mutagenesis.

A single-site saturation mutant library of NLuc was prepared using degenerate PCR primers in which the degenerate codon NNK (N indicates any nucleotide; K represents G or T) was substituted for each amino acid position from V2 through D11 of NLuc. In addition, a multisite saturation mutagenesis library was prepared in which all codons from V2 to W12 were replaced by NNK. PCR products were digested with EcoRI and KpnI enzymes, ligated into the similarly digested pXDC61, and then transformed into chemically competent E. coli NEB-5α. Representative plasmid clones were sequenced to identify specific amino acid substitutions, and select clones were transformed into L. pneumophila for translocation assays.

### Bacterial two-hybrid assay.

The bacterial two-hybrid system (BACTH) was used to investigate protein-protein interactions between NLuc and *Legionella* Icw and Dot proteins. The readout of BATCH relies on the interaction of one partner protein fused with bacteriophage λ CI interacting with a second protein fused with the α-subunit of E. coli RNA polymerase, as previously described (28). Colocalization of λ *c*I and α RNP through binding together of the protein partners activates transcription of a *lacZ* reporter containing an upstream λ *c*I-binding site. This system includes two plasmids, pACλCI, encoding λCI protein, and pBRα, encoding the RNA polymerase α subunit protein under the control of the lac *UV5* promoter. Using primers designed to insert amplified proteins into the NotI and BamHI restriction sites of each plasmid, the *icmW*, *icmS*, and *dotF* open reading frames were cloned into the multiple-cloning site of pACλCI. NLuc, NLuc16 missing the 15 N-terminal amino acids of NLuc, RalF, and SidD genes were cloned into the multicloning site of pBRα plasmid. Indicated plasmid pairs were introduced into reporter strain FW102-O_L_2-62 for readout of *lacZ* reporter expression, quantified in Miller units (58), after induction of protein expression with the indicated IPTG concentration. The fusion of λ *c*I with the physiologically nonrelevant β-flap subunit of E. coli RNA polymerase was used as a negative control.

### Macrophage infection and translocation assays.

One day before infection, the murine macrophage cell line J774A.1 (American Type Culture Collection, Manassas, VA) or macrophages transfected with GFP (as described below) were cultured in RPMI medium (Fisher Scientific, Waltham, MA) supplemented with 9% iron-supplemented calf serum (Gemini Bio-Products, West Sacramento, CA) and seeded into white 96-well or clear 6-well tissue culture plates in the same medium. L. pneumophila grown on BCYE plates with selection for expression vector (5 μg/mL of chloramphenicol) and thymidine (100 μg/mL) was harvested by resuspending in saline, centrifugation at 9,300 × *g* for 2 min to pellet bacteria, followed by washing twice with PBS. The bacterial cells were resuspended in RPMI supplemented with 100 μg/mL thymidine and used to infect J774A.1 cells at a MOI of 1 for 24 h or an MOI of 10 for 6 h at 37°C in 5% CO_2_, as specified, in the presence of 0.5 mM IPTG to induce expression of constructs.

At the indicated time points, infected J774A.1 cells were washed two times with PBS and lysed in PBS containing 0.2% saponin on ice for 1 h. The lysates were then centrifuged at 13,400 × *g* for 15 min. Supernatants, containing eukaryotic cytoplasm, were assayed for translocated NLuc activity using furimazine according to the manufacturer’s instructions or by Western blotting as described below. For one-step NLuc assays, infected J774A.1 cells in a 100-μL well volume were lysed by addition of 50 μL PBS containing 0.6% saponin and furimazine or the indicated coelenterazine analog. Luminescence or green fluorescence (485 nm excitation, 510 nm emission wavelengths) resulting from *in vivo* assembly of superfolder GFP was measured on a TECAN Infinite M1000 PRO plate reader.

For β-lactamase translocation assays, the β-lactamase (TEM-1) gene was fused to the N terminus of the LegA3 or LepA gene, or the C terminus of NLuc in pXDC61. *Legionella*, containing reporter plasmids, was grown on BCYE plates with 0.5 mM IPTG for 48 h to induce fusion gene expression. Bacteria were then washed with PBS, resuspended in RPMI medium with thymidine and IPTG, and used to infected to J774A.1 cells at an MOI of 50 as previously described (41). After 2 h, J774A.1 cells were loaded with 20 μL of 6× CCF4-AM solution (LiveBLAzer-FRET B/G loading kit, Invitrogen) containing 10 mM probenecid (Fisher Scientific). Loaded cells were incubated for 2 h at room temperature in the dark. Fluorescence was measured with an Infinite M1000 PRO plate reader (TECAN) with excitation at 409 nm and emission detected at both 460 nm and 530 nm.

### GFPopt-expressing J774A.1 cells.

Gryphon packaging cells (Allele Biotech, San Diego, CA) were transfected with 10 μg of pRetroX-GFPopt DNA using Lipofectamine LTX (ThermoFisher Scientific, Waltham, MA). After 3 days, supernatant was collected and used for transfection of J774A.1 cells. After a 3-day incubation, cells were replated in 6-well plates with RPMI medium containing 1 mg/mL G-418 (Sigma-Aldrich, St. Louis, MO). As described previously (57), individual colonies expressing GFP-opt were expanded in RPMI containing 500 μg/mL G-418 until use in experiments.

### Western blotting.

After infection for the indicated times, J774A.1 lysates were pelleted by centrifugation at 13,400 × *g* for 15 min at 4°C and denatured at 95°C for 5 min in SDS-PAGE sample buffer containing 5% β-mercaptoethanol. Bacterial pellets or saponin extracts from equivalent cell numbers were separated by SDS-PAGE. After electrophoresis, proteins were transferred to a nitrocellulose membrane (Bio-Rad, Hercules, CA). Membranes were blocked with 5% nonfat dried milk in Tris-buffered saline plus 0.1% Tween 20 (TBST), incubated in primary and secondary antibodies diluted in 5% nonfat dried milk in TBST for 1 h each, and then washed with TBST. Primary antibodies (murine monoclonal anti-FLAG M2 antibody, Sigma-Aldrich catalog number F1804; goat anti-GFP, Rockland Immunochemicals [Limerick, PA] catalog number 600-101-215; and mouse monoclonal anti-NanoLuc antibody, Promega catalog number N7000) and secondary antibodies [goat anti-mouse IgG (H+L) cross-adsorbed secondary antibody, horseradish peroxidase (HRP), ThermoFisher catalog number 31432; and rabbit anti-Goat IgG (H+L) secondary antibody, HRP, ThermoFisher catalog number 31402] were used at a 1:1,000 or 1:5,000 dilutions, respectively. Western blot signals were visualized by chemiluminescence using the SuperSignal West Femto maximum sensitivity substrate (ThermoFisher Scientific) and a ChemiDoc system (Bio-Rad, Hercules, CA).

### Confocal microscopy.

To observe *in vivo* assembly of sfGFP in J774A.1, GFPopt-expressing J774A.1 cells were plated in 12-well plates containing 1.5-thickness 12-mm round coverslips (Warner Instruments, Hamden, CT) at a density of 1 × 10^5^ cells per well. After 24 h, J774A.1 cells were infected with L. pneumophila expressing GFP11, GFP11::pRalF, or NLuc::GFP11 at an MOI of 1. Plates were immediately centrifuged at 930 × *g* for 10 min and then incubated at 37°C with 5% CO_2_. At 24 h postinfection, coverslips were fixed with 4% formaldehyde in PBS for 30 min at room temperature, permeabilized with 0.3% Triton X-100 in PBS for 15 min, mounted in ProLong Gold with 4',6-diamidino-2-phenylindole (DAPI; Invitrogen, Carlsbad, CA), and visualized with an LSM 880 confocal microscope (Carl Zeiss Microscopy, White Plains, NY) using enhanced GFP and DAPI settings for image collection. Image analysis was performed using Zen 2.1 software.

### Membrane permeability index determination.

L. pneumophila Lp02 was grown overnight in 2-aminoethanesulfonic acid-buffered yeast extract broth supplemented with 100 μg/mL thymidine, washed twice with PBS, and resuspended in PBS at ~1E8 bacteria/mL. A 1.0-μL volume of each coelenterazine substate (resuspended in 250 μg/mL of ethanol) was added to 1 mL of PBS or RIPA cell lysis buffer. Fifty microliters of substrate solution and 50 μL of bacteria were combined in 96-well black microtiter plates for luminescence detection in a TECAN Infinite M1000 PRO microplate reader. Bacterial cell permeability was calculated based on the ratio of luminescence for bacteria resuspended in PBS compared to luminescence of bacteria suspended in RIPA buffer. Data were normalized to results obtained for purified nanoluciferase (Nanolight Technologies, Pinetop, AZ), at 20 ng per assay well, to account for effects of RIPA buffer on luciferase activity.

### Statistics.

Statistical analysis was performed using Prism 9 for macOS (GraphPad, San Diego, CA) and methods indicated in text or figure panels, with a *P* level of <0.05 considered significant.
