# Supplementary material for: A Versatile Nanoluciferase Reporter Reveals Structural Properties Associated with a Highly Efficient, N-Terminal Legionella pneumophila Type IV Secretion Translocation Signal
Source: Microbiol Spectr. 2023 Feb 23;11(2):e02338-22. doi: 10.1128/spectrum.02338-22 (PMC10100965; doi:10.1128/spectrum.02338-22)
Supplement: Supplemental file 1 — Supplemental material. Download spectrum.02338-22-s0001.pdf, PDF file, 2.6 MB [file spectrum.02338-22-s0001.pdf]

# Supplemental Materials

**A Versatile Nanoluciferase Reporter Reveals Structural Properties Associated With a Highly Efficient, N-Terminal *Legionella pneumophila* type IV Secretion Translocation Signal**

Running title: Nanoluciferase T4SS translocation

Yoon-Suk Kang[1,2] and James E. Kirby[1,2 *]

[1]Department of Pathology, Beth Israel Deaconess Medical Center, Boston, MA

[2]Harvard Medical School, Boston, MA

## Supplementary Table of Contents

Supplementary Tables S1-S5, pages 3-17

Supplementary Figures S1-S5, pages 18-22

Supplemental References, page 23

**Table S1. Effects of Single Amino Acid Substitutions in V2 through D11 of Nluc on Luciferase Activity and Translocation.**

| | Luminescence intensity[a, b] | | Translocation efficiency[a] | | Average translocation efficiency | Normalized translocation[c] |
|---|---|---|---|---|---|---|
| NLuc wt Lp02fla (dot/icm+) | 1.00 | 1.00 | 1.00 | 1.00 | 1.00 | 100% |
| Nluc wt Lp03fla (*dotA-*) | 1.21 | 1.05 | 0.08 | 0.16 | 0.12 | 0% |
| V2Lys | 2.04 | 1.69 | 1.43 | 1.48 | 1.45 | 151% |
| V2Ala | 0.51 | 1.42 | 1.23 | 1.40 | 1.31 | 136% |
| V2Ser | 0.29 | 0.54 | 0.83 | 0.88 | 0.86 | 84% |
| V2Trp | 0.74 | 0.61 | 1.42 | 1.55 | 1.48 | 155% |
| V2Gly | 1.17 | 0.98 | 1.27 | 1.47 | 1.37 | 142% |
| V2Asn | 1.49 | 1.24 | 0.57 | 1.39 | 0.98 | 98% |
| V2His | 4.72 | 1.93 | 1.42 | 1.28 | 1.35 | 140% |
| V2Phe | 2.22 | 1.85 | 1.30 | 1.43 | 1.37 | 142% |
| V2Ile | 0.00 | 0.03 | 0.65 | 1.24 | 0.94 | 94% |
| V2Met | 1.11 | 0.93 | 1.23 | 1.36 | 1.30 | 134% |
| V2Tyr | 3.60 | 1.40 | 1.25 | 1.30 | 1.28 | 132% |
| V2Glu | 0.20 | 0.57 | 2.35 | 1.71 | 2.03 | 217% |
| V2Arg | 0.52 | 0.83 | 1.66 | 1.63 | 1.65 | 174% |
| V2Asp | 0.18 | 0.35 | 2.59 | 1.55 | 2.07 | 222% |
| V2Cys | 1.14 | 0.95 | 1.14 | 1.27 | 1.21 | 124% |
| V2Pro | 0.02 | 0.12 | 1.03 | 1.47 | 1.25 | 129% |
| V2Thr | 2.71 | 1.26 | 1.18 | 1.31 | 1.25 | 128% |
| V2Leu | 2.79 | 1.33 | 1.19 | 1.16 | 1.17 | 120% |
| V2Gln | 1.16 | 0.96 | 1.32 | 1.24 | 1.28 | 132% |
| F3Ala | 1.67 | 1.09 | 0.55 | 1.16 | 0.85 | 83% |
| F3Ile | 2.07 | 1.12 | 1.11 | 1.72 | 1.42 | 147% |
| F3Asn | 2.51 | 1.29 | 0.57 | 0.50 | 0.54 | 47% |
| F3Glu | 1.26 | 1.05 | 0.66 | 1.02 | 0.84 | 82% |
| F3Gln | 0.72 | 0.80 | 0.41 | 1.28 | 0.84 | 82% |
| F3Met | 1.45 | 1.20 | 1.26 | 1.06 | 1.16 | 118% |
| F3Thr | 1.21 | 0.81 | 1.12 | 1.32 | 1.22 | 125% |
| F3Pro | 2.76 | 1.29 | 0.84 | 1.19 | 1.01 | 102% |
| F3Leu | 2.34 | 1.34 | 0.84 | 1.45 | 1.14 | 116% |
| F3Ser | 2.00 | 1.27 | 0.45 | 1.06 | 0.75 | 72% |
| F3Tyr | 1.65 | 0.97 | 1.07 | 1.32 | 1.20 | 122% |
| F3His | 8.13 | 2.77 | 1.12 | 0.96 | 1.04 | 105% |
| F3Lys | 1.57 | 1.31 | 0.98 | 1.05 | 1.02 | 102% |

| | | | | | | |
|---|---|---|---|---|---|---|
| F3Arg | 0.86 | 1.71 | 0.61 | 0.96 | 0.78 | 75% |
| F3Cys | 3.82 | 1.18 | 0.53 | 0.67 | 0.60 | 55% |
| F3Val | 2.28 | 1.30 | 0.35 | 0.50 | 0.43 | 35% |
| F3Gly | 1.78 | 1.48 | 0.44 | 0.59 | 0.52 | 45% |
| F3Trp | 3.02 | 1.32 | 0.37 | 0.52 | 0.45 | 37% |
| F3Asp | 5.10 | 2.25 | 0.44 | 0.76 | 0.60 | 55% |
| T4Gly | 0.74 | 1.62 | 1.05 | 1.19 | 1.12 | 114% |
| T4Pro | 1.56 | 1.30 | 0.86 | 1.46 | 1.16 | 118% |
| T4Val | 1.51 | 1.25 | 0.82 | 0.83 | 0.82 | 80% |
| T4Cys | 0.01 | 0.10 | 1.32 | 1.01 | 1.16 | 118% |
| T4Leu | 1.58 | 1.21 | 0.83 | 0.97 | 0.90 | 88% |
| T4Ser | 0.87 | 0.72 | 1.07 | 0.98 | 1.03 | 103% |
| T4Trp | 7.10 | 2.91 | 1.30 | 1.45 | 1.37 | 142% |
| T4Asn | 9.61 | 3.00 | 1.42 | 1.43 | 1.42 | 148% |
| T4Lys | 9.75 | 3.11 | 1.44 | 1.57 | 1.50 | 157% |
| T4Gln | 0.70 | 0.58 | 1.09 | 1.24 | 1.16 | 119% |
| T4Asp | 2.53 | 1.61 | 1.45 | 1.33 | 1.39 | 145% |
| T4Arg | 4.87 | 2.05 | 1.18 | 1.20 | 1.19 | 122% |
| T4Met | 5.77 | 2.80 | 1.78 | 1.60 | 1.69 | 179% |
| T4Tyr | 6.85 | 2.70 | 1.47 | 1.12 | 1.29 | 133% |
| T4His | 1.34 | 1.11 | 0.88 | 1.03 | 0.95 | 95% |
| T4Phe | 1.24 | 1.03 | 0.90 | 0.92 | 0.91 | 90% |
| T4Ile | 0.60 | 0.80 | 0.98 | 1.22 | 1.10 | 111% |
| T4Ala | 1.17 | 0.98 | 1.08 | 1.09 | 1.09 | 110% |
| T4Glu | 0.98 | 0.82 | 1.58 | 1.12 | 1.35 | 140% |
| L5Arg | 0.00 | 0.02 | 1.38 | 1.28 | 1.33 | 138% |
| L5Gly | 1.25 | 1.04 | 0.76 | 0.63 | 0.69 | 65% |
| L5Trp | 1.58 | 1.32 | 1.23 | 1.15 | 1.19 | 122% |
| L5Thr | 1.56 | 1.30 | 1.64 | 1.52 | 1.58 | 166% |
| L5Gln | 1.48 | 1.13 | 0.96 | 0.83 | 0.90 | 88% |
| L5Glu | 1.44 | 1.10 | 0.86 | 0.84 | 0.85 | 83% |
| L5Ala | 1.12 | 0.94 | 0.82 | 0.84 | 0.83 | 80% |
| L5Lys | 1.58 | 1.32 | 2.82 | 1.28 | 2.05 | 220% |
| L5Ser | 0.08 | 0.04 | 1.23 | 1.52 | 1.37 | 143% |
| L5Cys | 1.06 | 0.88 | 0.82 | 1.15 | 0.99 | 98% |
| L5Asp | 0.05 | 0.10 | 1.01 | 1.01 | 1.01 | 102% |
| L5Asn | 0.44 | 0.37 | 0.89 | 1.02 | 0.95 | 95% |
| L5Phe | 0.07 | 0.06 | 0.89 | 0.91 | 0.90 | 89% |
| L5Ile | 3.24 | 1.70 | 1.06 | 1.09 | 1.08 | 109% |
| L5Met | 1.74 | 1.25 | 1.21 | 1.11 | 1.16 | 118% |
| L5Val | 0.15 | 0.06 | 0.86 | 0.76 | 0.81 | 78% |
| L5His | 0.06 | 0.15 | 0.85 | 0.98 | 0.92 | 91% |
| L5Tyr | 0.09 | 0.07 | 0.99 | 1.24 | 1.11 | 113% |

| | | | | | | |
|---|---|---|---|---|---|---|
| L5Pro | 0.35 | 0.19 | 0.83 | 0.88 | 0.86 | 84% |
| E6Cys | 3.24 | 1.69 | 1.37 | 0.94 | 1.16 | 118% |
| E6Val | 0.39 | 0.82 | 0.82 | 0.57 | 0.70 | 66% |
| E6Gly | 0.20 | 0.97 | 0.38 | 0.50 | 0.44 | 36% |
| E6Trp | 1.28 | 1.26 | 0.93 | 0.76 | 0.85 | 82% |
| E6Arg | 1.53 | 1.27 | 1.02 | 0.66 | 0.84 | 82% |
| E6Pro | 1.72 | 1.13 | 0.87 | 0.51 | 0.69 | 64% |
| E6Gln | 0.59 | 1.49 | 1.01 | 0.65 | 0.83 | 81% |
| E6Leu | 0.75 | 1.62 | 1.16 | 0.81 | 0.99 | 99% |
| E6Ser | 2.68 | 1.23 | 1.07 | 0.73 | 0.90 | 89% |
| E6Ala | 0.13 | 0.81 | 0.69 | 0.36 | 0.52 | 46% |
| E6Ile | 0.86 | 0.92 | 0.96 | 0.63 | 0.80 | 77% |
| E6His | 0.76 | 1.64 | 0.79 | 0.46 | 0.63 | 57% |
| E6Met | 0.89 | 0.94 | 1.33 | 0.99 | 1.16 | 118% |
| E6Lys | 4.70 | 0.91 | 1.03 | 0.68 | 0.86 | 84% |
| E6Tyr | 3.84 | 1.19 | 1.36 | 1.01 | 1.19 | 121% |
| E6Thr | 0.79 | 1.66 | 1.34 | 0.98 | 1.16 | 118% |
| E6Asn | 3.59 | 0.99 | 1.47 | 1.10 | 1.29 | 133% |
| E6Asp | 2.99 | 1.49 | 1.00 | 0.63 | 0.82 | 79% |
| E6Phe | 2.57 | 1.14 | 1.14 | 0.77 | 0.95 | 95% |
| D7Thr | 3.40 | 1.83 | 0.67 | 0.54 | 0.61 | 55% |
| D7Trp | 1.75 | 1.06 | 0.54 | 0.54 | 0.54 | 47% |
| D7Arg | 0.45 | 0.78 | 0.38 | 0.41 | 0.40 | 31% |
| D7Cys | 2.35 | 1.05 | 0.85 | 0.68 | 0.77 | 73% |
| D7Ala | 3.12 | 1.90 | 0.54 | 0.72 | 0.63 | 58% |
| D7Leu | 0.54 | 0.75 | 0.46 | 0.41 | 0.44 | 36% |
| D7Asn | 2.28 | 1.39 | 1.52 | 1.40 | 1.46 | 153% |
| D7Gly | 1.17 | 0.98 | 0.55 | 0.50 | 0.52 | 46% |
| D7Val | 1.16 | 0.97 | 0.34 | 0.42 | 0.38 | 29% |
| D7Pro | 2.41 | 1.00 | 0.63 | 0.50 | 0.57 | 51% |
| D7Phe | 0.00 | 0.01 | 0.41 | 0.35 | 0.38 | 29% |
| D7Ile | 0.04 | 0.09 | 0.77 | 0.65 | 0.71 | 67% |
| D7Met | 0.18 | 0.05 | 0.48 | 0.36 | 0.42 | 34% |
| D7Ser | 0.00 | 0.03 | 0.42 | 0.49 | 0.46 | 38% |
| D7Tyr | 0.00 | 0.01 | 0.46 | 0.54 | 0.50 | 43% |
| D7His | 0.32 | 0.16 | 0.46 | 0.34 | 0.40 | 31% |
| D7Gln | 1.55 | 0.29 | 0.53 | 0.41 | 0.47 | 40% |
| D7Lys | 1.23 | 1.02 | 0.81 | 0.79 | 0.80 | 77% |
| D7Glu | 0.00 | 0.01 | 0.50 | 0.37 | 0.44 | 36% |
| F8Arg | 0.00 | 0.01 | 1.89 | 1.55 | 1.72 | 182% |
| F8Ser | 0.02 | 0.01 | 0.86 | 0.66 | 0.76 | 73% |
| F8Pro | 0.01 | 0.01 | 1.34 | 1.37 | 1.36 | 141% |
| F8Gly | 0.02 | 0.03 | 0.88 | 1.10 | 0.99 | 99% |

| | | | | | | |
|---|---|---|---|---|---|---|
| F8Gln | 0.01 | 0.04 | 0.99 | 0.76 | 0.87 | 86% |
| F8Ala | 0.26 | 0.12 | 0.91 | 0.58 | 0.74 | 71% |
| F8Thr | 0.28 | 0.13 | 0.90 | 0.92 | 0.91 | 90% |
| F8Leu | 0.21 | 0.18 | 1.11 | 0.77 | 0.94 | 93% |
| F8Ile | 0.21 | 0.19 | 0.93 | 0.96 | 0.94 | 93% |
| F8Met | 0.18 | 0.15 | 1.19 | 1.26 | 1.22 | 126% |
| F8Tyr | 1.96 | 1.16 | 1.04 | 0.81 | 0.92 | 91% |
| F8His | 0.00 | 0.01 | 0.76 | 0.78 | 0.77 | 74% |
| F8Asn | 0.00 | 0.02 | 0.83 | 0.60 | 0.72 | 68% |
| F8Lys | 0.00 | 0.01 | 1.13 | 0.90 | 1.01 | 102% |
| F8Asp | 0.00 | 0.02 | 0.93 | 0.60 | 0.76 | 73% |
| F8Glu | 0.00 | 0.02 | 1.13 | 1.16 | 1.14 | 116% |
| F8Cys | 0.04 | 0.01 | 1.02 | 1.23 | 1.13 | 114% |
| F8Trp | 0.34 | 0.18 | 1.04 | 0.70 | 0.87 | 85% |
| F8Val | 0.63 | 0.53 | 0.98 | 1.01 | 1.00 | 100% |
| V9Ser | 3.00 | 1.49 | 1.44 | 1.31 | 1.37 | 143% |
| V9Asp | 2.78 | 1.31 | 1.23 | 1.10 | 1.17 | 119% |
| V9Glu | 1.34 | 1.12 | 0.99 | 1.04 | 1.02 | 102% |
| V9Gly | 0.00 | 0.08 | 2.16 | 1.80 | 1.98 | 211% |
| V9Phe | 0.80 | 0.27 | 0.96 | 0.60 | 0.78 | 75% |
| V9Thr | 5.25 | 2.37 | 1.46 | 1.10 | 1.28 | 132% |
| V9Cys | 1.31 | 1.69 | 1.78 | 1.36 | 1.57 | 165% |
| V9Pro | 0.64 | 0.73 | 1.54 | 1.18 | 1.36 | 141% |
| V9Arg | 1.46 | 1.02 | 1.72 | 1.36 | 1.54 | 162% |
| V9Leu | 0.97 | 0.50 | 1.00 | 0.86 | 0.93 | 92% |
| V9Ile | 2.16 | 1.30 | 0.97 | 1.02 | 0.99 | 99% |
| V9Met | 1.44 | 1.20 | 0.82 | 0.90 | 0.86 | 83% |
| V9Ala | 1.61 | 1.04 | 0.88 | 0.52 | 0.70 | 66% |
| V9Tyr | 0.00 | 0.01 | 0.64 | 0.72 | 0.68 | 63% |
| V9His | 0.52 | 0.13 | 0.76 | 0.82 | 0.79 | 76% |
| V9Gln | 3.85 | 2.21 | 0.87 | 0.73 | 0.80 | 77% |
| V9Asn | 0.66 | 0.55 | 1.31 | 0.95 | 1.13 | 115% |
| V9Lys | 0.04 | 0.04 | 0.91 | 0.99 | 0.95 | 95% |
| V9Trp | 0.44 | 0.17 | 1.01 | 0.65 | 0.83 | 81% |
| G10Ala | 4.32 | 1.60 | 1.08 | 0.95 | 1.01 | 102% |
| G10Arg | 0.15 | 0.12 | 0.89 | 0.56 | 0.73 | 69% |
| G10Cys | 1.45 | 1.20 | 1.00 | 1.05 | 1.03 | 103% |
| G10Phe | 4.03 | 1.36 | 1.24 | 1.11 | 1.18 | 120% |
| G10Leu | 0.43 | 0.35 | 0.99 | 1.23 | 1.11 | 112% |
| G10Trp | 0.25 | 0.11 | 0.86 | 0.53 | 0.69 | 65% |
| G10Glu | 0.55 | 0.35 | 1.24 | 1.48 | 1.36 | 141% |
| G10Val | 0.17 | 0.19 | 1.04 | 1.09 | 1.06 | 107% |
| G10Ile | 0.01 | 0.03 | 0.93 | 0.79 | 0.86 | 84% |

| | | | | | | |
|---|---|---|---|---|---|---|
| G10Met | 0.02 | 0.03 | 0.90 | 1.14 | 1.02 | 102% |
| G10Ser | 0.38 | 0.12 | 0.86 | 0.92 | 0.89 | 87% |
| G10Pro | 0.00 | 0.03 | 0.79 | 1.19 | 0.99 | 98% |
| G10Thr | 0.00 | 0.01 | 0.96 | 0.81 | 0.88 | 87% |
| G10Tyr | 0.00 | 0.02 | 0.80 | 0.94 | 0.87 | 85% |
| G10His | 0.01 | 0.02 | 0.71 | 0.93 | 0.82 | 79% |
| G10Gln | 0.02 | 0.03 | 0.89 | 0.85 | 0.87 | 85% |
| G10Asn | 0.11 | 0.04 | 0.88 | 1.19 | 1.03 | 104% |
| G10Lys | 0.00 | 0.02 | 0.76 | 0.93 | 0.85 | 82% |
| G10Asp | 0.58 | 0.48 | 1.13 | 0.81 | 0.97 | 97% |
| D11Glu | 5.07 | 3.22 | 1.44 | 1.22 | 1.33 | 138% |
| D11Lys | 6.68 | 3.56 | 0.54 | 0.73 | 0.63 | 58% |
| D11Ala | 5.17 | 2.30 | 0.76 | 0.58 | 0.67 | 62% |
| D11Met | 0.69 | 0.57 | 0.95 | 1.29 | 1.12 | 114% |
| D11Thr | 3.31 | 2.75 | 0.91 | 1.11 | 1.01 | 101% |
| D11Arg | 4.75 | 2.95 | 0.62 | 0.82 | 0.72 | 68% |
| D11Cys | 6.62 | 3.51 | 1.06 | 1.18 | 1.12 | 114% |
| D11Gly | 4.35 | 3.62 | 1.07 | 1.14 | 1.11 | 112% |
| D11Leu | 4.48 | 2.73 | 1.20 | 0.88 | 1.04 | 104% |
| D11Phe | 0.00 | 0.00 | 1.08 | 1.01 | 1.05 | 105% |
| D11Ile | 0.01 | 0.00 | 0.95 | 0.81 | 0.88 | 86% |
| D11Ser | 0.01 | 0.01 | 1.07 | 1.33 | 1.20 | 123% |
| D11Pro | 0.00 | 0.02 | 1.00 | 0.88 | 0.94 | 93% |
| D11Val | 0.00 | 0.00 | 0.92 | 1.27 | 1.10 | 111% |
| D11Tyr | 0.00 | 0.00 | 1.08 | 1.26 | 1.17 | 120% |
| D11His | 0.00 | 0.03 | 1.04 | 0.85 | 0.95 | 94% |
| D11Gln | 0.07 | 0.10 | 0.87 | 1.20 | 1.03 | 104% |
| D11Asn | 0.67 | 0.56 | 1.00 | 1.28 | 1.14 | 116% |
| D11Trp | 0.13 | 0.21 | 0.98 | 0.85 | 0.92 | 91% |

[a]replicates are from two independent experiments (biological replicates), each infection with the indicated NLuc mutant performed in a well of a 6-well tissue culture plate.

[b]Luminescence readings are from bacterial pellets after saponin extraction of eukaryotic cytoplasm.

[c]normalized to translocation of wild type Nluc observed in dot/icm$^{+}$ and *dotA*$^{-}$ strains set at 100% and 0%, respectively.

# Table S2. RaptorX Predicted Secondary Structure of the N-termini of Wild Type and Mutated Versions of Nanoluciferase.

| mutation | sequence | ss3 prediction | ss8 prediction | solvent accessibility | relative translocation[a] | relative luminescence |
|---|---|---|---|---|---|---|
| >wildtype | MVFTLEDFVGDWRQTAGYNLDQVLEQ | CEEEHHHHHHHHHHHCCCCHHHHHCC | LEEEHHHHHHHHHHHHTLLHHHHHLL | EEMEMEEEEEEEEEEEEEEEEEEEEE | 1 | 1 |
| >delta6E | MVFTLDFVGDWRQTAGYNLDQVLEQG | CEEEEEEECCHHHHCCCHHHHHHHCC | LEEEEEEEELHHHHTTLLHHHHHHLL | EEMEBEMEEEEEEEEEMEEEEEEEEE | 0.24 | 0.99 |
| >delta7D | MVFTLEFVGDWRQTAGYNLDQVLEQG | CEEEEEEECCHHHCCCCCHHHHHHCC | LEEEEEEEELHHHHTTLLHHHHHHLL | EEMEBEMMEEEEEEEEMEEEEEEEEE | 0.22 | 1.0 |
| >delta6E7D | MVFTLFVGDWRQTAGYNLDQVLEQGG | CEEEEEECCHHHCCCCCHHHHHHCCC | LEEEEEEELHHHHTTLLHHHHHHHLL | EEMEBMMMEMEEEEMEEEEEBEEEEE | 0.24 | 1.15 |
| >E6GD7A | MVFTLGAFVGDWRQTAGYNLDQVLEQ | CEEEHHHHHHHHHHHCCCCHHHHHCC | LEEEHHHHHHHHHHHTTLLHHHHHLL | EEMEEEEEEEEEEEEEEEEEEEEEEE | 0.65 | 1.05 |
| >E6G | MVFTLGDFVGDWRQTAGYNLDQVLEQ | CEEEHHHHHHHHHHHCCCCHHHHHCC | LEEEHHHHHHHHHHHTTLLHHHHHLL | EEMEEEEEEEEEEEEEEEEEEEEEEE | 0.72 | 1.04 |
| >D7A | MVFTLEAFVGDWRQTAGYNLDQVLEQ | CEEEHHHHHHHHHHHCCCCHHHHHCC | LEEEHHHHHHHHHHHHTLLHHHHHLL | EEMEEEEEEEEEEEEEEEEEEEEEEE | 0.72 | 0.99 |
| >E6GD11A | MVFTLGDFVGAWRQTAGYNLDQVLEQ | CEEEHHHHHHHHHHHCCCCHHHHHCC | LEEEHHHHHHHHHHHHTLLHHHHHLL | EEMEEEEEEEEEEEEEEEEEEEEEEE | 0.65 | 1.04 |
| >E6GD7AD11A | MVFTLGAFVGAWRQTAGYNLDQVLEQ | CEEEHHHHHHHHHHHCCCCHHHHHCC | LEEEHHHHHHHHHHHHTLLHHHHHLL | EEEEEEEEEEEEEEEEEEEEEEEEEE | 0.65 | 1.07 |
| >V2W | MWFTLEDFVGDWRQTAGYNLDQVLEQ | CEEEHHHHHHHHHHHCCCCHHHHHCC | LEEEHHHHHHHHHHHHTLLHHHHHLL | EEMEMEEEEEEEEEEEEEEEEEEEEE | 1.08 | 5.78 |
| >V2WE6Q | MWFTLQDFVGDWRQTAGYNLDQVLEQ | CEEEHHHHHHHHHHHCCCCHHHHHCC | LEEEHHHHHHHHHHHHTLLHHHHHLL | EEMEEEEEEEEEEEEEEEEEEEEEEE | 0.87 | 4.11 |
| >V2WE6TV9G | MWFTLTDFGGDWRQTAGYNLDQVLEQ | CEEEEECCCCCCHHCCCCHHHHHHCC | LEEEEEEELLLHHHHTTLLHHHHHLL | EEMEMEEEEEEEEEEEEMEEEEEEEE | 0.2 | 2.39 |
| >E6QD11G | MVFTLQDFVGGWRQTAGYNLDQVLEQ | CEEEHHHHHHCHHHCCCCCHHHHHCC | LEEEHHHHHHHHHHHTTLLHHHHHLL | EEMEMEEEEEEEEEEEEEEEEEEEEE | 0.99 | 8.91 |
| >E6QD11R | MVFTLQDFVGRWRQTAGYNLDQVLEQ | CEEEHHHHHHHHHHHCCCCHHHHHCC | LEEEHHHHHHHHHHHHTLLHHHHHLL | EEMEEEEEEEEEEEEEEEEEEEEEEE | 1 | 9.94 |
| >V2WV9T | MWFTLEDFTGDWRQTAGYNLDQVLEQ | CEEEHHHHHCCHHHHCCCCHHHHHCC | LEEEHHHHHHHHHHHTTLLHHHHHLL | EEMEMEEEEEEEEEEEEEEEEEEEEE | 1.2 | 9.25 |
| >D11R | MVFTLEDFVGRWRQTAGYNLDQVLEQ | CEEEHHHHHHHHHHHCCCCHHHHHCC | LEEEHHHHHHHHHHHHTLLHHHHHLL | EEMEMEEEEEEEEEEEEEEEEEEEEE | 1.31 | 7.5 |
| >D11T | MVFTLEDFVGTWRQTAGYNLDQVLEQ | CEEEHHHHHHHHHHHCCCCHHHHHCC | LEEEHHHHHHHHHHHTTLLHHHHHLL | EEMEMEEEEEEEEEEEEEEEEEEEEE | 0.83 | 5.94 |
| >D11G | MVFTLEDFVGGWRQTAGYNLDQVLEQ | CEEEHHHHHHHHHHHCCCCHHHHHCC | LEEEHHHHHHHHHHHTTLLHHHHHLL | EEMEMEEEEEEEEEEEEEEEEEEEEE | 0.86 | 4.68 |
| >F3GE6QD11G | MVGTLQDFVGGWRQTAGYNLDQVLEQ | CCEEHHHHHHHHHHHCCCCHHHHHCC | LLEEHHHHHHHHHHHTLLLHHHHHLL | EEEEEEEEEEEEEEEEEEEEEEEEEE | 1.03 | 2.86 |
| >E6T | MVFTLTDFVGDWRQTAGYNLDQVLEQ | CEEEHHHHHHHHHHHCCCCHHHHHCC | LEEEHHHHHHHHHHHTTLLHHHHHLL | EEMEMEEEEEEEEEEEEEEEEEEEEE | 0.56 | 1.84 |
| >V2WE6TV9G | MWFTLTDFGGDWRQTAGYNLDQVLEQ | CEEEEECCCCCCHHCCCCHHHHHHCC | LEEEEEEELLLHHHHTTLLHHHHHLL | EEMEMEEEEEEEEEEEEMEEEEEEEE | 0.2 | 2.39 |
| >V2WE6TD7TV9G | MWFTLTTFGGDWRQTAGYNLDQVLEQ | CEEEEEEECCCCCCCCCCHHHHHHCC | LEEEEEEEELLHHHHTTLLHHHHHLL | EEMEMEEEEEEEEEEEEMEEEEEEEE | 0.23 | 1.93 |
| >V2WE6GV9G | MWFTLGDFGGDWRQTAGYNLDQVLEQ | CEEEECCCCCCHHHCCCCHHHHHHCC | LEEEEELLLLLHHHHTTLLHHHHHLL | EEMEEEEEEEEEEEEEEMEEEEEEEE | 0.21 | 1.96 |
| >V2WV9G | MWFTLEDFGGDWRQTAGYNLDQVLEQ | CEEEECCCCCCHHHCCCCHHHHHHCC | LEEEEEEELLLHHHHTTLLHHHHHLL | EEMEMEEEEEEEEEEEEMEEEEEEEE | 1.04 | 1.59 |
| >T4QW12Q | MVFQLEDFVGDQRQTAGYNLDQVLEQ | CCECHHHHHHHHHHHCCCCHHHHHCC | LLELHHHHHHHHHHHHTLLHHHHHLL | EEMEEEEEEEEEEEEEEEEEEEEEEE | 1 | 1.34 |
| >T4SD11GW12V | MVFSLEDFVGGVRQTAGYNLDQVLEQ | CEEEHHHHHHHHHHHCCCCHHHHHCC | LEEEHHHHHHHHHHHTLLLHHHHHLL | EEMEEEEEEEEEEEEEEEEEEEEEEE | 0.99 | 1.93 |
| >F3QG10R | MVQTLEDFVRDWRQTAGYNLDQVLEQ | CCEEHHHHHHHHHHHHCCCHHHHHCC | LLEEHHHHHHHHHHHHTLLHHHHHLL | EEEEEEEEEEEEEEEEEEEEEEEEEE | 1.03 | 1.45 |
| >F3LG10TW12R | MVLTLEDFVTDRRQTAGYNLDQVLEQ | CEEEHHHHHHHHHHHCCCCHHHHHCC | LEEEHHHHHHHHHHHHTLLHHHHHLL | EEMEEEEEEEEEEEEEEEEEEEEEEE | 1.03 | 1.15 |
| >T4WG10N | MVFWLEDFVNDWRQTAGYNLDQVLEQ | CEEEHHHHHHHHHHHCCCCHHHHHCC | LEEEHHHHHHHHHHHHTLLHHHHHLL | EEEEMEEEEEEEEEEEEEEEEEEEEE | 1.05 | 1.28 |
| >V2DW12R | MDFTLEDFVGDRRQTAGYNLDQVLEQ | CCCCHHHHHHHHHHHCCCCHHHHHCC | LLLLHHHHHHHHHHHTTLLHHHHHLL | EEMEEEEEEEEEEEEEEEEEEEEEEE | 0.98 | 2.3 |
| >V2DD11Q | MDFTLEDFVGQWRQTAGYNLDQVLEQ | CCCCHHHHHHHHHHHCCCCHHHHHCC | LLLLHHHHHHHHHHHHTLLHHHHHLL | EEMEEEEEEEEEEEEEEEEEEEEEEE | 0.86 | 1.87 |
| >F8SG10R | MVFTLEDSVRDWRQTAGYNLDQVLEQ | CEEEHHHHHHHHHHHHCCCHHHHHCC | LEEEHHHHHHHHHHHHTLLHHHHHLL | EEMEEEEEEEEEEEEEEEEEEEEEEE | 0.89 | 1.36 |
| >F8WW12V | MVFTLEDWVGDVRQTAGYNLDQVLEQ | CEEEHHHHHHHHHHHCCCCHHHHHCC | LEEEHHHHHHHHHHHHTLLHHHHHLL | EEMEMEEEEEEEEEEEEEEEEEEEEE | 1.11 | 1.09 |

| | | | | | | |
|---|---|---|---|---|---|---|
| >F8WW12N | MVFTLEDWVGDNRQTAGYNLDQVLEQ | CEEEHHHHHCCCCCCCCCCHHHHHCC | LEEEHHHHHLLHHHLLLLLHHHHHLL | EEMEMEEEEEEEEEEEEEEEEEEEE | 0.71 | 0.86 |
| >F8SG10Q | MVFTLEDSVQDWRQTAGYNLDQVLEQ | CEEEHHHHHHHHHHHHCCCHHHHHCC | LEEEHHHHHHHHHHHHHTLLHHHHHLL | EEMEEEEEEEEEEEEEEEEEEEEEE | 1.21 | 1.12 |
| >T4SF8Q | MVFSLEDQVGDWRQTAGYNLDQVLEQ | CEEEHHHHHHHHHHHCCCCHHHHHCC | LEEEHHHHHHHHHHHHHTLLHHHHHLL | EEMEEEEEEEEEEEEEEEEEEEEEE | 0.66 | 1.43 |
| >F3DT4S | MVDSLEDFVGDWRQTAGYNLDQVLEQ | CCCCHHHHHHHHHHHCCCCHHHHHCC | LLLLHHHHHHHHHHHHTLLHHHHHLL | EEEEEEEEEEEEEEEEEEEEEEEEE | 1.1 | 1.31 |
| >D11RW12R | MVFTLEDFVGRRRQTAGYNLDQVLEQ | CEEEHHHHHHHHHHHCCCCHHHHHCC | LEEEHHHHHHHHHHHTLLLHHHHHLL | EEMEEEEEEEEEEEEEEEEEEEEEE | 1.17 | 1.13 |
| >V2RD7VW12R | MRFTLEVFVGDRRQTAGYNLDQVLEQ | CCEEEHHHHCCHHHCCCCCHHHHHCC | LEEEEHHHHHLHHHHTTLLHHHHHLL | EEMEBEEEEEEEEEEEEEEEEEEEE | 1.09 | 0.87 |
| >V2WD11RW12Q | MWFTLEDFVGRQRQTAGYNLDQVLEQ | CEEEHHHHHHHHHHHCCCCHHHHHCC | LEEEHHHHHHHHHHHHLLLHHHHHLL | EEMEEEEEEEEEEEEEEEEEEEEEE | 1.23 | 1.26 |
| >V2WE6G | MWFTLGDFVGDWRQTAGYNLDQVLEQ | CCCCHHHHHHHHHHHCCCCHHHHHCC | LLLLHHHHHHHHHHHHTLLHHHHHLL | EEBEBEEBEEEMEEEEEMEMEEBEEE | 0.85 | 0.9 |
| >E6GV9G | MVFTLGDFGGDWRQTAGYNLDQVLEQ | CEEEECCCCCCCCCCCCCCHHHHHCC | LEEEELLLLLLHHHHHLLLHHHHHLL | EMBBBBEBEEEMEEEBEMEBEEBMEE | 0.23 | 1.0 |

[a]Normalized to translocation of wild type NLuc expressed in a dot/icm$^{+}$ strain without subtraction of background signal observed during infection with *dotA*$^{-}$ controls.

**Table S3. Relative permeability and luminesce of commercially available furimazine analogs.**

| analog | relative permeability[a] | relative luminesence[b] |
|---|---|---|
| furimazine | 3.1±0.3 | 1 |
| coelenterazine | 1.9±0.4 | 0.12 |
| 400a | 0.6±0.1 | 7.7 |
| cp | 0.4±0.1 | 0.09 |
| e | 1.3±0.1 | 0.62 |
| e-F | 3.3±0.4 | 0.69 |
| f | 4.9±0.3 | 0.21 |
| fcp | 1.0±0.1 | 0.53 |
| h | 0.4±0.1 | 2.8 |
| hcp | 5.3±0.7 | 0.12 |
| i | 0.7±0.0 | 1.6 |
| ip | 0.2±0.1 | 0.02 |
| n | 0.5±0.0 | 2.5 |
| v | 1.0±0.1 | 0.01 |

[a]relative permeability index was calculated as the ratio of luminescence from bacteria suspended in PBS to the luminescence of bacteria permeabilized in radioimmunoprecipitation (RIPA) buffer containing 1% NP-40 and 1% sodium deoxycholate. Results were normalized to activity of purified nanoluciferase added to PBS and RIPA alone, as RIPA buffer variably inhibited or stimulated activity of different nanoluciferase substrates up to 10-fold. The relative permeability ratios are the mean of values obtained from two independent experiments each with two technical replicates.

[b]luminescence signal relative to furimazine substrate based on testing an equivalent amount of dot/icm$^{+}$ bacteria expressing nanoluciferase permeabilized in RIPA buffer, i.e., the denominator in the relative permeability index.

**Table S4. Bacterial strains and plasmids**

| Strain or plasmid | Relevant markers and characteristics | Reference |
|---|---|---|
| **Bacteria**: | | |
| *Legionella pneumophila* | | |
| Lp02*flaA* | Philadelphia 1, *thyA rpsL hsdR flaA* | |
| Lp03*flaA* | *thyA rpsL hsdR dotA03 flaA,* Dot/Icm translocation deficient | (1) |
| Lp02 | Philadelphia 1, *thyA rpsL hsdR* | (2) |
| Lp03 | Dot/Icm translocation deficient, *thyA rpsL hsdR* | (2) |
| *ΔicmW* | Lp02, *icmW* | (3) |
| *ΔicmS* | Lp02, *icmS* | Vogel Lab. |
| *ΔicmW ΔicmS* | Lp02, *icmW icmS* | Vogel Lab. |
| *ΔlvgA* | Lp02, *lvgA* | Vogel Lab. |
| *Escherichia coli* | | |
| NEB-5α | *fhuA2 Δ(argF-lacZ)U169 phoA glnV44 Φ80 Δ(lacZ)M15 gyrA96 recA1 relA1 endA1 thi-1 hsdR17* | NEB |
| FW102 $O_L$2-62 | FW102 containing an F' Kan bearing the p*lac*$O_L$2–62-*lacZ* fusion where the λCI operator is centered at position –62 upstream of the *lac* promoter, $Km^R$ | (4) |
| **Eukaryote**: | | |
| J774a.1 | Mouse macrophage, ATCC TIB-67 | ATCC |
| Gryphon | Retroviral packaging cell line | Allele Biotechnology |
| **Plasmid**: | | |
| pRetroX-Tet-Off Advanced | Retroviral vector for mammalian expression, $Neo^R$ or $Km^R$ | Clontech |
| pRetroX-GFPopt | pRetroX vector expressing GFPopt | This study |
| pXDC61 | pMMB207C *ΔmobA, blaM*, $Cam^R$ | (5) |
| pXDC61-NLuc | pXDC61 expressing Nano Luciferase | This study |
| pXDC61-3FNLuc | Nano Luciferase having 3xFLAG on N'-terminus | This study |
| pXDC61-NLuc3F | Nano Luciferase having 3xFLAG on C'-terminus | This study |
| pXDC61-3FNLuc::RalF | Translational fusion with naoluciferase and whole RalF | This study |
| pXDC61-3FNLuc::pRalF | Protein fusion with nanoluciferase and partial RalF | This study |
| pXDC61-NLuc10N | Nanoluciferase deleting 10 AA on N'-terminus | This study |
| pXDC61-NLuc30N | Nanoluciferase deleting 30 AA on N'-terminus | This study |
| pXDC61-NLuc10C | Nanoluciferase deleting 10 AA on C'-terminus | This study |
| pXDC61-pNLuc::TetR | Translational fusion with partial NLuc on N'-terminus of TetR | This study |

| | | |
|---|---|---|
| pXDC61-TetR::pNLuc | Translational fusion with partial NLuc on C'-terminus of TetR | This study |
| pXDC61-pNLuc::TEV | Translational fusion with partial NLuc on N'-terminus of TEV protease | This study |
| pXDC61-TEV::pNLuc | Translational fusion with partial NLuc on C'-terminus of TEV protease | This study |
| pXDC61-NLucE6 | Nanoluciferase deleted with 6$^{th}$ Glu | This study |
| pXDC61-NLucD7 | Nanoluciferase deleted with 7$^{th}$ Asp | This study |
| pXDC61-NLucE6D7 | Nanoluciferase deleted with 6$^{th}$ Glu & 7$^{th}$ Asp | This study |
| pXDC61-NLucV2X-D11X | NLuc site saturation mutant library, 2$^{nd}$ Val to 11$^{th}$ Asp | This study |
| pXDC61-GFP11::pRalF | Translational fusion with GFP11 and partial RalF on C'-terminus | This study |
| pXDC61-NLuc::GFP11 | Translational fusion with nanuciferase and GFP11 on C'-terminus | This study |
| pXDC61-GFP11:LegA3 | Fusion protein with LegA3 and GFP11 on N'-terminus | This study |
| pXDC61-GFP11:LepA | GFP11-LepA fusion protein | This study |
| pXDC61-GFP11:RalF | GFP11-whole RalF fusion protein | This study |
| pXDC61-GFP11 | GFP11 fragment | This study |
| pXDC61-TEM | β-lactamase TEM region | This study |
| pXDC61-NLuc:TEM | NLuc-TEM fusion protein | This study |
| pXDC61-TEM:LegA3 | TEM-LegA3 fusion protein | This study |
| pXDC61-TEM:LepA | TEM-LepA fusion protein | This study |
| pBRα-β flap (831-1057) | Contains p*lac*UV5- and p*lpp* promoters directeing synthesis of the α-subunit of RNAP fused via three alanines to residues 831–1057 of the β subunit of *E. coli* RNAP, Amp$^R$ | (4) |
| pBRα-IcmW | Fused with *L. pneumophila* IcmW | This study |
| pBRα-IcmS | Fused with *L. pneumophila* IcmS | This study |
| pBRα-DotF | Fused with *L. pneumophila* DotF | This study |
| pACλCI-β flap (831-1057) | Contains p*lac*UV5 promoter directing synthesis of λCI fused via three alanines to residues 831–1057 of the β subunit of *E. coli* RNAP, Cam$^R$ | (4) |
| pACλCI-NLuc | Fused with NLuc | This study |
| pACλCI-NLuc16 | Fused with NLuc deleting 15 AA on N-terminus | This study |
| pACλCI-RalF | Fused with Lp RalF | This study |
| pACλCI-SidD | Fused with Lp SidD | This study |

NEB: New England Biolabs; ATCC: American Type Culture Collection; Neo: neomycin; Km: kanamycin; Cam: Chloramphenicol; Amp: Ampicillin

**Table S5. Primers used in this study**

| Primer name | Sequence 5'-3'* | Resulting constructs |
|---|---|---|
| NLucEcoRI-f | CGC **GAATTC** ATG GTC TTC ACA CTC GAA GAT | pXDC61-NLuc |
| NLucKpnI-r | CGC **GGTACC** CGC CAG AAT GCG TTC GCA CAG | |
| 3FNLucEcoRI-f | CGC **GAATTC** ATG GAC TAC AAA GAC CAT GAC GGT GAT TAT AAA GAT CAT GAC ATC GAT TAC AAG GAT GAC GAT GAC AAG GTC TTC ACA CTC GAA GAT TTC | pXDC61-3FNLuc |
| 3FNLucKpnI-r | CGC **GGTACC** TTA CTT GTC ATC GTC ATC CTT GTA ATC GAT GTC ATG ATC TTT ATA ATC ACC GTC ATG GTC TTT GTA GTC CGC CAG AAT GCG TTC GCA CAG | pXDC61-NLuc3F |
| RalF_KpnI-f | CGC **GGTACC** GAC TAC AAA GAC CAT GAC | pXDC61-3FNLuc::RalF |
| RalF_BamHI-r | CGC **GGATCC** TTA AAA TTT TAA TTG TCT | |
| RalF355-f | CGC **GGTACC** GGC GTT CCC AAA GAT CCA GAC | pXDC61-3FNLuc::pRalF |
| RalF_BamHI-f | CGC **GGATCC** ATG CAT CCA GAA ATT GAA | pCYA-RalF |
| RalF_SalI-r | CGC **GTCGAC** TTA AAA TTT TAA TTG TCT | |
| NLucN10-f | CGC **GAATTC** ATG GAC TAC AAA GAC CAT GAC GGT GAT TAT AAA GAT CAT GAC ATC GAT TAC AAG GAT GAC GAT GAC AAG GAC TGG CGA CAG ACA GCC GGC | pXDC61-NLuc10N |

| | | |
|---|---|---|
| NLucN30-f | CGC **GAATTC** ATG GAC TAC AAA GAC CAT GAC GGT GAT TAT AAA GAT CAT GAC ATC GAT TAC AAG GAT GAC GAT GAC AAG AGT TTG TTT CAG AAT CTC GGG | pXDC61-NLuc30N |
| NLucC10-r | CGC **GGTACC** TTA CAT TCC GTT GAT GGT TAC TCG | pXDC61-NLuc10C |
| pNLucTetR-f | CGC **GAATTC** TAA GGA GGA AAA AAA ATG GTC TTC ACA CTC GAA GAT TTC | pXDC61-pNLuc::TetR |
| pNLucTetR-r | CGC **GGTACC** TTA CCC GGG GAG CAT GTC AAG GTC | |
| TetRpNLuc-f | CGC **GAATTC** TAA GGA GGA AAA AAA ATG GGC AGC TCT AGA CTG GAC AAG AGC AAA | pXDC61-TetR::pNLuc |
| TetRpNLuc-r | CGC **GGTACC** TTA GGA CAC ACC TCC CTG TTC AAG GAC | |
| pNLucTEV-f | CGC **GAATTC** TAA GGA GGA AAA AAA ATG GTC TTC ACA CTC GAA GAT TTC GTT | pXDC61-pNLuc::TEV |
| pNLucTEV-r | CGC **GGTACC** TTA GTT CAT TAA CTG TGT CGC TTC | |
| TEVpNLuc-f | CGC **GAATTC** TAA GGA GGA AAA AAA ATG GGG GAG AGT CTG TTT AAA GGC | pXDC61-TEV::pNLuc |
| TEVpNLuc-r | CGC **GGTACC** TTA GGA CAC ACC TCC CTG TTC AAG | |
| NLucE6-f | CGC **GAATTC** TAA GGA GGA AAA AAA ATG GTC TTC ACA CTC GAT TTC GTT | pXDC61-NLucE6 |
| NLucD7-f | CGC **GAATTC** TAA GGA GGA AAA AAA ATG GTC TTC ACA CTC GAG TTC GTT | pXDC561-NLucD7 |
| NLucE6D7-f | CGC **GAATTC** TAA GGA GGA AAA AAA ATG GTC TTC ACA CTC TTC GTT GGG | pXDC61-NLucE6G/D7 A |
| NLucV2X -f | CGC **GAATTC** TAA GGA GGA AAA AAA ATG NNK TTC ACA CTC GAA | pXDC61-NLucV2X |

| | | |
|---|---|---|
| NLucF3X-f | CGC **GAATTC** TAA GGA GGA AAA AAA ATG GTC NNK ACA CTC GAA | pXDC61-NLucF3X |
| NLucT4X-f | CGC **GAATTC** TAA GGA GGA AAA AAA ATG GTC TTC NNK CTC GAA | pXDC61-NLucT4X |
| NLucL5X-f | CGC **GAATTC** TAA GGA GGA AAA AAA ATG GTC TTC ACA NNK GAA GAT | pXDC61-NLucL5X |
| NLucE6X-f | CGC **GAATTC** TAA GGA GGA AAA AAA ATG GTC TTC ACA CTC NNK GAT TTC | pXDC61-NLucE6X |
| NLucD7X-f | CGC **GAATTC** TAA GGA GGA AAA AAA ATG GTC TTC ACA CTC GAA NNK TTC GTT | pXDC61-NLucD7X |
| NLucF8X-f | CGC **GAATTC** TAA GGA GGA AAA AAA ATG GTC TTC ACA CTC GAA GAT NNK GTT GGG | pXDC61-NLucF8X |
| NLucV9X-f | CGC **GAATTC** TAA GGA GGA AAA AAA ATG GTC TTC ACA CTC GAA GAT TTC NNK GGG GAC | pXDC61-NLucV9X |
| NLucG10X-f | CGC **GAATTC** TAA GGA GGA AAA AAA ATG GTC TTC ACA CTC GAA GAT TTC GTT NNK GAC TGG | pXDC61-NLucG10X |
| NLucD11X-f | CGC **GAATTC** TAA GGA GGA AAA AAA ATG GTC TTC ACA CTC GAA GAT TTC GTT GGG NNK TGG | pXDC61-NLucD11X |
| NLucR2-12-f | CGC **GAATTC** TAA GGA GGA AAA AAA ATGNNKNNKNNKNNKNNKNNKNNKNNKNNKNNKNNKT GGCGACAGACA | Multi-site SSM |
| | | |
| GFPopt-f | CGC **GGATCC** TCGCCACC ATG AGC AAA GGA GAA GAA CTT | pRetroX-GFPopt |
| GFPopt-r | CGC **GAATTC** CTA CTT TTC GTT GGG ATC TTT C | |

| | | |
|---|---|---|
| GFP11pRalF-f | CGC **GAATTC** TAA GGA GGA AAA AAA ATG CGT GAC CAC ATG GTC CTT | pXDC61-GFP11::pRalF |
| GFP11pRalF-r | CGC **GGTACC** TTA AAA TTT TAA TTG TCT | |
| NLucGFP11-f | CGC **GAATTC** TAA GGA GGA AAA AAA ATG GTC TTC ACA CTC GAA GAT | pXDC61-NLuc::GFP11 |
| NLucGFP11-r | CGC **GGTACC** TTA TGT AAT CCC AGC AGC ATT | |
| GFP11-f | CGC **GAATTC** TAA GGA GGA AAA AAA ATG CGT GAC CAC ATG GTC CTT | pXDC61-GFP11 |
| TEM-f | CGC **GAATTC** TAA GGA GGA AAA AAA ATG GCT CAC CCA GAA ACG CTG | pXDC61-TEM |
| TEM-r | CGC **GGTACC** TTA CCA ATG CTT AAT CAG TGA G | |
| NLucTEM-f | CGC **GAATTC** TAA GGA GGA AAA AAA ATGGTCTTCACACTCGAA | pXDC61-NLuc:TEM |
| NLucTEM-r | CGC **GGTACC** CGC CAG AAT GCG TTC GCA | |
| LegA3-f | CGC **GGTACC** GGT GGT GGC GGC TCC AGT ATT GCA AAC GAT ATT ATC AGT | pXDC61-TEM:LegA3 or pXDC61-GFP11:LegA3 |
| LegA3-r | CGC **TCTAGA** TTA TAG GCC TGT CGC AAC | |
| LepA-f | CGC **GGTACC** GGT GGT GGC GGC TCC CTA TTT GCA AGA AGG | pXDC61-TEM:LepA or pXDC61-GFP11:LepA |
| LepA-r | CGC **TCTAGA** CTA CTT TTT TTT ATC GTT TGA | |

| | | |
|---|---|---|
| GFP11RalF-f | CGC **GGTACC** GGTGGTGGCGGCTCC CAT CCA GAA ATT GAA AAA | pXDC61-GFP11:RalF |
| GFP11RalF-r | CGC **TCTAGA** TTA AAA TTT TAA TTG TCT ACC TT | |
| NLucNotI-f | CGC **GCGGCCGC**A GTC TTC ACA CTC GAA GAT | pBRα-NLuc |
| NLuc16NotI-f | CGC **GCGGCCGC**A GCC GGC TAC AAC CTG GAC | pBRα-NLuc16 |
| NLucBamHI-r | CGC **GGATCC** TTA CGC CAG AAT GCG TTC | |
| IcmWNotI-f | CGC **GCGGCCGC**A CCT GAT TTA AGC CAT GAA | pACλCI-IcmW |
| IcmWBamHI-r | CGC **GGATCC** TTA TTC ATC CCC TTC GAG | |
| IcmSNotI-f | CGC **GCGGCCGC**A GAG CGA GAT ATT AGC AAG | pBRα-IcmS or pACλCI-IcmS |
| IcmSBamHI-r | CGC **GGATCC** CTA ATC ATA CAT TAA CTC | |
| DotFNotI-f | CGC **GCGGCCGC**A ATG GCA GAG CAC GAT CAA | pACλCI-DotF |
| DotFBamHI-r | CGC **GGATCC** TCA ACT ATC TTC TTG ACT | |
| RalFNotI-f | CGC **GCGGCCGC**A CAT CCA GAA ATT GAA AAA | pBRα-RalF |
| RalFBamHI-r | CGC **GGATCC** TTA AAA TTT TAA TTG TCT | |
| SidDNotI-f | CGC **GCGGCCGC**A GTA TAT TAT GAG ATC ATT | pBRα-SidD |
| SidDBamHI-r | CGC **GGATCC** TTA AAT AGT AAG ACT CGA | |

* Bold with underlining indicates restriction endonuclease sites. Also, underlined NNK sequences indicate positions of amino acid substitution used in creation of the NLuc-SSM libraries.

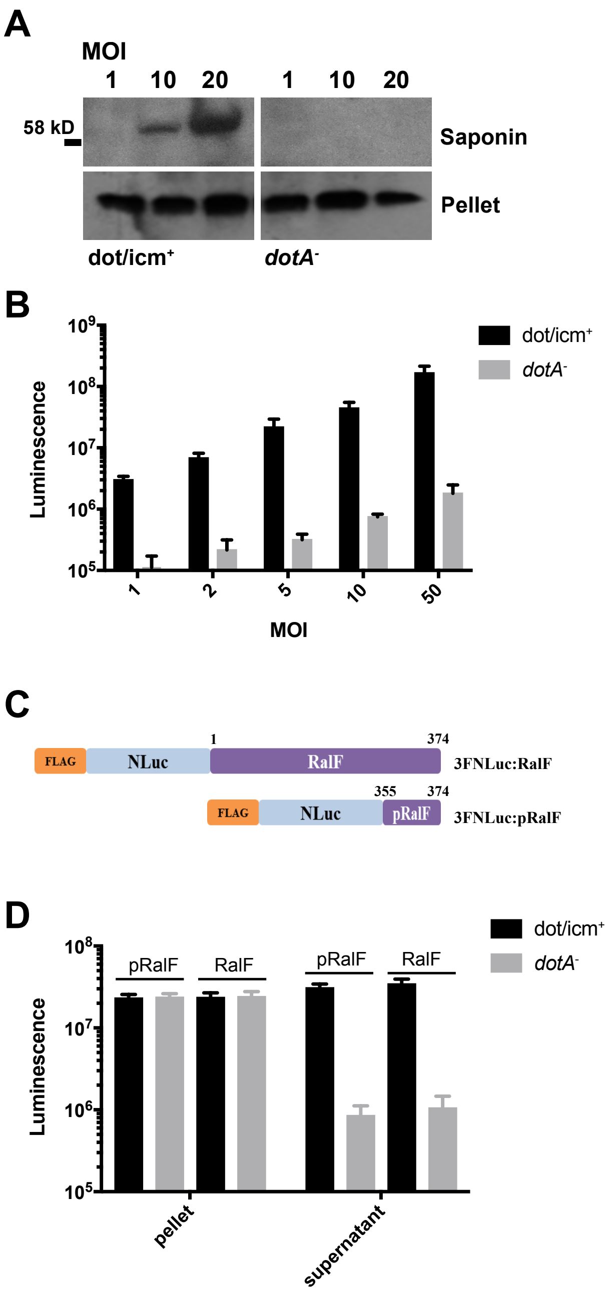


**Figure S1. Translocation of nanoluciferase:RalF fusion proteins into J774A.1 macrophages.** Macrophages were infected with different multiplicities of infection (MOIs) of wild-type (wt), dot/icm$^+$ (Lp02 *flaA*$^-$) or *dotA*$^-$ T4SS-incompetent (Lp03 *flaA*$^-$) *L. pneumophila* expressing an 3xFlag(3F):NLuc:RalF protein fusion. **(A)** Western blot detection of translocated 3F:NLuc:RalF fusion protein with anti-FLAG tag antibody 6 h post infection. Translocated protein is found in the extracted eukaryotic cytoplasm (supernatant), while bacterial-associated, untranslocated protein remains in the bacterial pellet. **(B)** Detection of translocated luciferase activity under similar conditions. **(C)** Diagram of NLuc constructs fused with whole RalF (amino acids 1-374) or partial RalF (C-terminal amino acids, 355-374), designated NLuc:RalF or NLuc:pRalF, respectively. **(D)** Translocation of NLuc:RalF and NLuc:pRalF expressed in a *dot/icm*$^+$ or *dotA*$^-$ strain following a 6 h infection at an MOI of 10. Mean luminescence and standard deviation from three independent experiments are shown.

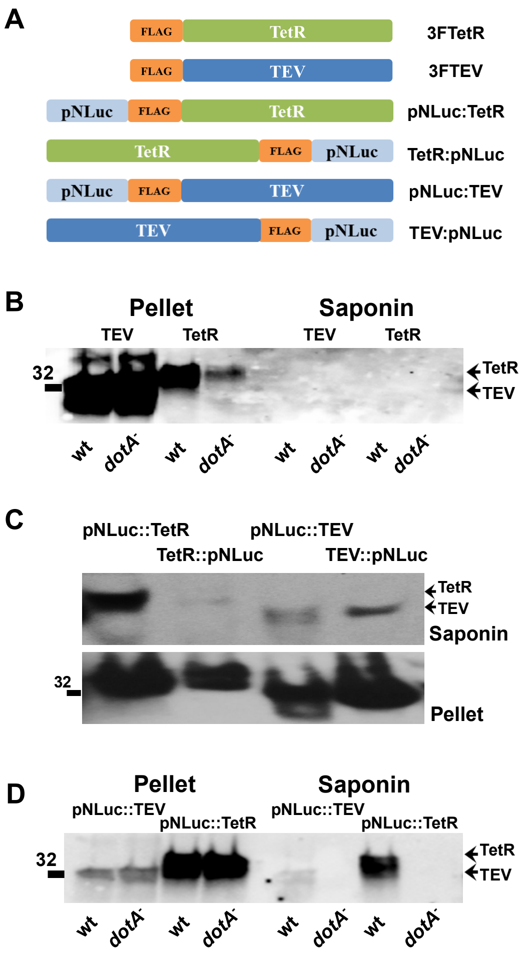


**Figure S2. The N-terminus of NLuc is necessary and sufficient for T4SS-dependent translocation. (A)** Structure of fusions of the 30 N-terminal amino acids of NLuc (pNluc) to the N- and C-terminus of TetR and TEV protease proteins, respectively, with incorporation of a 3X-FLAG epitope to enable detection by western blot. Fusions of 3X-FLAG to the N-terminus of TetR and TEV in the absence of NLuc were used as controls. **(B)** Western blots of J774A.1 cells infected with dot/icm$^+$ (wt) or *dotA*$^-$ strains at an MOI of 10 in strains expressing TEV and TetR proteins with 3X FLAG at the N-terminus in the absence of an NLuc translocation signal. At 6 h post infection, eukaryotic cytoplasm extract and insoluble pellet were separated by SDS-PAGE, and western blots probed with anti-FLAG tag antibody. TEV and TetR were only detected in the pellet fraction, indicating absence of an intrinsic translocation signal in these proteins. **(C)** Infections with strains expressing 3F-TEV or 3F-TetR fused to the first 30 amino acids of NLuc (pNluc) at the C- or N-terminus. All versions of TEV and TetR fused with pNLuc showed translocation into eukaryotic cytoplasm (supernatant), with the efficiencies of N-terminal and C-terminal fusions differing by protein partner. **(D)** Translocation only occurred when fusion proteins were expressed in a T4SS-competent *dot/icm*$^+$ (wt), but not in T4SS-incompetent, *dotA*$^-$ strain.

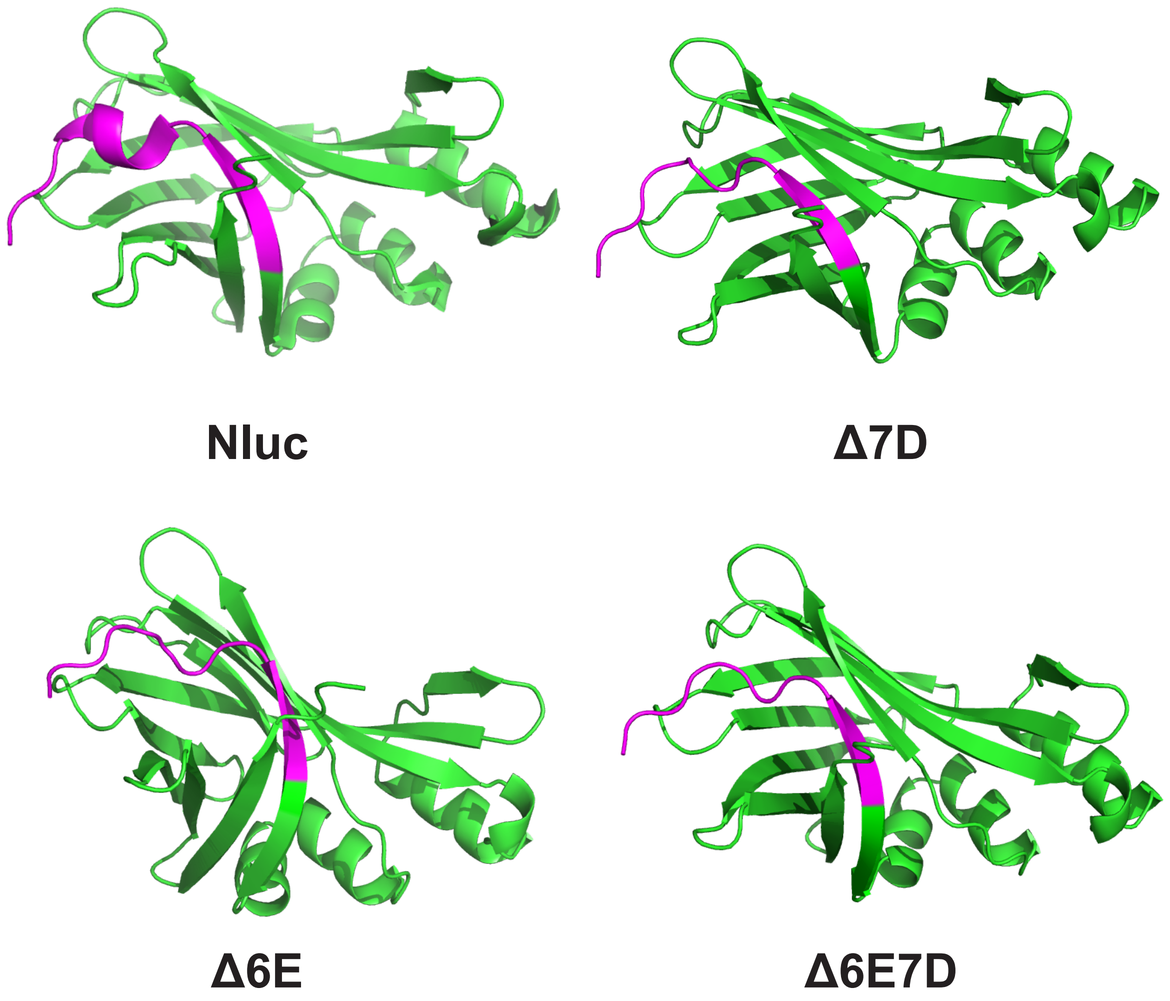


**Figure S3. AlphaFold2 predictions of mutant NLuc structures.** AlphaFold2 CoLab (6) predictions of the structure of NLuc, NLucΔ6E, NLucΔ7D, and NLucΔ6EΔ7D. The surface-exposed, N-termini (M1-W12) are colored magenta. In the three deletion mutants, the N-terminal α-helix, present in the structure predicted by AlphaFold2 and the solved structure of NLuc (PDB 5IBO), is now an unstructured region.

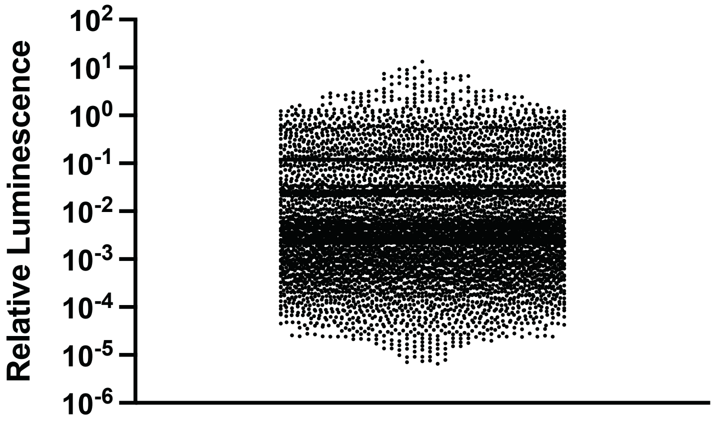


**Figure S4. Luminescence associated with random mutagenesis of the N-terminus of NLuc.** Approximately seven thousand random mutants of the NLuc N-terminus, inclusive of amino acids V2 to W12, were generated using a degenerate, primer randomization strategy and were tested for preservation of luminescence when expressed in *E. coli K-12*. Shown are the luminescence of analyzed clones normalized to wild type NLuc. Approximately 30 clones with preserved and/or elevated luminescence were further tested in *L. pneumophila* for T4SS-dependent translocation, as described in the main body of the manuscript.

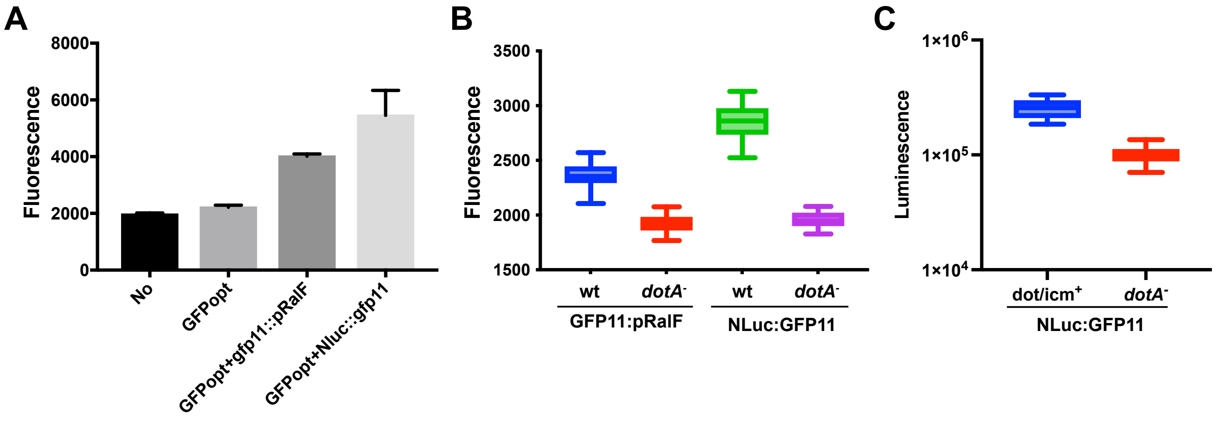


**Figure S5. Development of a split GFP high throughput T4SS assay using an NLuc translocation signal. (A)** The non-fluorescent GFP11 fragment and GFPopt protein can combine to yield fluorescent superfolder GFP (sfGFP)**.** To determine whether this assembly remained functional when T4SS translocation signals were attached to the small GFP11 fragment, the fluorescent signal from combination of plasmids expressing GFP11 fused with NLuc (GFP11:NLuc) or partial RalF (GFP11:pRalF) and GFPopt were co-expressed in *E. coli* K-12. Bars represent the mean and standard deviation for three independent experiments performed in duplicate after overnight incubation and induction of protein expression with 0.5 mM IPTG. **(B)** The ability to use this system in a high throughput screening format to quantify T4SS-dependent translocation was assessed by infecting J774A.1 cells expressing GFPopt with *L. pneumophila* strains producing GFP11:pRalF or NLuc:GFP11. Fluorescent intensity was assayed in white 96-well plates; and combined data from two independent experiments, each performed with 40 technical replicates, are shown as box and whisker plots (median bar; boxed 25th and 75th percentiles; and whiskers, range). Data were used to determine the Z' described in the results section. **(C)** NLuc:GFP11 also provided a single-step luciferase readout in combination with coelenterazine h. Box and whisker plots represent the combined data from two independent experiments, each performed with ten technical replicates. Notably, through infecting J774A.1 GFPopt host cells with bacteria expressing NLuc:GFP11, the same NLuc:GFP11 construct can provide orthogonal sfGFP fluorescence- and nanoluciferase-based translocation readouts in the same high throughput screening wells, thereby presumably significantly reducing the burden of time-consuming secondary assays for screening hit confirmation.

# References


1. Coers J, Vance RE, Fontana MF, Dietrich WF. 2007. Restriction of Legionella pneumophila growth in macrophages requires the concerted action of cytokine and Naip5/Ipaf signalling pathways. Cell Microbiol 9:2344-57.
2. Berger KH, Isberg RR. 1993. Two distinct defects in intracellular growth complemented by a single genetic locus in Legionella pneumophila. Mol Microbiol 7:7-19.
3. Zuckman DM, Hung JB, Roy CR. 1999. Pore-forming activity is not sufficient for Legionella pneumophila phagosome trafficking and intracellular growth. Mol Microbiol 32:990-1001.
4. Dove SL, Joung JK, Hochschild A. 1997. Activation of prokaryotic transcription through arbitrary protein-protein contacts. Nature 386:627-30.
5. de Felipe KS, Glover RT, Charpentier X, Anderson OR, Reyes M, Pericone CD, Shuman HA. 2008. Legionella eukaryotic-like type IV substrates interfere with organelle trafficking. PLoS Pathog 4:e1000117.
6. Mirdita M, Schütze K, Moriwaki Y, Heo L, Ovchinnikov S, Steinegger M. 2022. ColabFold - Making protein folding accessible to all. bioRxiv doi:10.1101/2021.08.15.456425:2021.08.15.456425.
